# Genome-Wide Characterization and Expression Analysis of the *nsLTP* Gene Family in Potato (*Solanum tuberosum*) in Response to Drought Stress

**DOI:** 10.3390/biology15211947

**Published:** 2026-10-28

**Authors:** Dan Wang, Mengyang Pan, Junxiang Mu, Yanhong Ma, Mengyuan Zhong, Zerong Zhang, Jiale Zhang, Yao Chen, Tuanrong Lin, Yiqian Wang, Longqiu Fan, Xinlei Jiao, Zhicheng Zhang

**Affiliations:** 1 College of Life Science and Technology, Jining Normal University, Ulanqab 012000, China; 2 College of Agriculture, Inner Mongolia Agricultural University, Hohhot 010000, China; 3 Potato Research Laboratory, Ulanqab Academy of Agricultural and Forestry Sciences, Ulanqab 012000, China; 4 Institute of Biotechnology, Inner Mongolia Academy of Science and Technology, Hohhot 010000, China

**Keywords:** potato, *nsLTP*, phylogeny, drought stress, bioinformatics, expression analysis

## Abstract

Potato (*Solanum tuberosum* L.) is the fourth largest food crop in the world, and drought stress seriously affects its yield and quality. Plant non-specific lipid transfer proteins (nsLTPs) play an important role in resisting biotic and abiotic stresses. Although the functions of nsLTPs have been reported in crops such as rice (*Oryza sativa* L.) and *Arabidopsis thaliana*, the systematic identification and functional analysis of nsLTPs in potato, a key food crop sensitive to drought, remain very scarce. In this study, the whole genome identification, drought stress expression analysis and potential function prediction of the potato *StnsLTP* gene family were carried out by bioinformatics and real-time quantitative polymerase chain reaction (RT-qPCR). A total of 123 potato *StnsLTP* genes were identified, which were unevenly distributed on 11 chromosomes. The encoded amino acid length was 88–390 aa, and 91.1% of StnsLTP proteins were hydrophobic. Conserved motif analysis showed that motif 4 was the most important conserved motif in this family. There were 0–4 introns and 1–4 exons. The family identified 45 pairs of tandem duplicated genes and 20 pairs of segmentally duplicated genes, and the *nsLTP* potato genes showed close phylogenetic relationships with tomato *nsLTP* genes. Combined with transcriptome data, three PEG-responsive candidate genes, *StnsLTP.115*, *StnsLTP.117*, and *StnsLTP.118*, were screened. After polyethylene glycol 6000 (PEG 6000) simulated drought treatment, the three genes showed different degrees of induced expression in the roots, stems and leaves of the three potato cultivars, and their expression differed markedly across cultivars, tissues and sampling time points. This study systematically elucidated the structure and evolutionary characteristics of the potato *StnsLTP* family, and identified three candidate genes responsive to PEG-simulated drought stress. This not only provides a basic reference for the functional study of the potato *StnsLTP* family, but also offers clues for analyzing potato drought response pathways and exploring candidate genes for potato stress response research.

## Simple Summary

Potato is one of the most important food crops worldwide, and drought represents a major environmental factor limiting potato production. Previous studies have revealed that non-specific lipid transfer proteins help plants cope with harmful environmental conditions. In this study, we systematically analyzed the gene family encoding these proteins in potato across the whole genome and explored how these genes change their activity under drought conditions. We found several genes that can react to drought stress by using biological sequence analysis and gene expression testing. These findings deepen our understanding of the evolution and biological roles of these potato genes. The drought-responsive genes we identified provide valuable genetic resources to study potato drought resistance and breed new potato cultivars that can survive dry environments.

## 1. Introduction

Potato is a dicotyledonous species in the Solanaceae, native to the Andes Mountains of South America [1]. Characterized by high yield, wide adaptability, and rich nutrients such as starch, protein, minerals, vitamins and dietary fiber, potato ranks as the world’s fourth most important staple food crop after rice, wheat (*Triticum aestivum*), and maize (*Zea mays*), with a global production of 386.7 million tonnes in 2023 according to FAOSTAT. It plays an irreplaceable role in optimizing agricultural structure, improving farmers’ livelihoods, and supporting sustainable agricultural development [2,3]. Currently, China’s food security faces multiple challenges such as population growth, farmland reduction, and climate change, making the expansion of potato production strategically significant for ensuring food security and advancing rural revitalization [4]. However, global warming has exacerbated abiotic stresses including heat and drought, which severely restrict plant growth and development [5]. As a drought-prone country, China has arid and semi-arid regions accounting for 47% of its land area, with particularly severe drought stress in western China that limits local agricultural production [6]. In northern China, frequent droughts severely restrict potato growth during tuber formation and expansion, causing substantial yield losses [7]. As a shallow-rooted crop, potato is highly sensitive to drought, which causes wilting, restricts shoot and root growth, and disturbs physiological metabolism [8]. Especially in the tuber expansion period, drought will hinder the accumulation of dry matter, directly resulting in a significant decrease in yield and a significant deterioration in quality [9]. In recent years, significant progress has been made in potato stress-resistant genetic engineering, and the improved reference genome resources have laid a solid foundation for mining key drought-resistant genes and enhancing stress tolerance in major cultivars [10,11,12]. Lipids are essential for maintaining normal cellular physiological functions and regulating stress-related signaling pathways, thus playing key roles in plant growth, development, and stress responses [13]. Therefore, genetic engineering-mediated improvement of drought resistance is an effective strategy to achieve stable potato production.

Non-specific lipid transfer proteins (nsLTPs), also known as lipid transfer proteins (LTPs), belong to the PR-14 family of plant pathogenesis-related proteins. They are so named due to their ability to bind diverse lipid molecules and mediate the transport of phospholipids and fatty acids between membranes in vitro [14]. nsLTPs are widely distributed in terrestrial plants, ranging from lower bryophytes such as Marchantia to higher vascular plants, whereas no homologous genes have been identified in aquatic photosynthetic organisms such as algae [15]. The N-terminus of nsLTPs contains a signal peptide that mediates their secretion into the extracellular space. The mature protein harbors a conserved eight-cysteine motif (ECM) with the typical structure C-Xn-C-Xn-CC-Xn-CXC-Xn-C-Xn-C, which forms four intramolecular disulfide bonds to generate a stable hydrophobic cavity [16].

Studies have shown that *nsLTPs* play important roles in plant biotic and abiotic stress responses, signal transduction, cuticular wax biosynthesis, and seed development and reproduction [17]. With the rapid advances of whole-genome sequencing and high-throughput sequencing technologies, genome-wide association studies (GWAS) have been extensively applied to mine genetic loci and molecular markers for crop abiotic stress tolerance traits such as salt tolerance and drought tolerance. In species including rice, maize, sorghum (*Sorghum bicolor*), sunflower (*Helianthus annuus*), and mung bean (*Vigna radiata*), a large number of loci and candidate genes associated with stresses such as drought, salinity, low temperature, and heat stress have been successfully identified. GWAS has not only accelerated the discovery of stress-related genetic loci in diverse species, but also established a solid basis for dissecting the functions of stress-responsive gene families including *nsLTPs* [18,19,20,21,22]. Accumulating evidence indicates that *nsLTPs* are crucial regulators of plant drought tolerance. They alleviate drought-induced oxidative damage, enhance water-use efficiency, and maintain cellular homeostasis by stabilizing membranes, increasing antioxidant enzyme activities, promoting osmolyte accumulation, and inducing the expression of stress-responsive genes [23,24,25]. In potato, overexpression of *StnsLTP1* enhances cell membrane integrity, increases antioxidant enzyme activities and ascorbic acid content, and upregulates stress-responsive genes under multiple abiotic stresses [26]. Similarly, heterologous overexpression of chestnut *CmnsLTP6.9* in *Arabidopsis* enhances ROS-scavenging capacity and mitigates oxidative damage caused by drought, consistent with the conserved drought-regulating mechanism of *nsLTPs* [27]. This conserved regulatory mechanism is also widely present in other major crops. In kenaf (*Hibiscus cannabinus*), heterologous expression of *HcnsLTP111* significantly enhances drought tolerance, whereas gene silencing results in increased reactive oxygen species (ROS) accumulation, reduced antioxidant activity, and compromised osmotic adjustment [28]. In common bean (*Phaseolus vulgaris*), genome-wide analysis identified 58 *PvLTP* genes, nine of which were strongly induced by drought stress, implying their potential roles in drought adaptation [16]. In tobacco (*Nicotiana tabacum*), overexpression of *NtLTPI.38* enhances drought tolerance by reinforcing antioxidant defense, decreasing superoxide anion and hydrogen peroxide accumulation, and promoting lateral root development [29].

At present, the *nsLTP* gene family has been systematically identified in numerous plant species, including tomato (*Solanum lycopersicum*), *Arabidopsis*, sesame (*Sesamum indicum*), maize, rice, cotton (*Gossypium hirsutum*), and cabbage (*Brassica oleracea* var. *capitata*) [30,31,32,33,34,35,36]. Previous potato *nsLTP* studies used only a single-conserved-domain screening approach on the older genome assembly, failing to recover the full complement of family members and resulting in incomplete gene annotation. Compared with the older assembly, DM6.1 is a chromosome-level genome with improved contiguity and refined gene models, which reduces fragmented gene predictions and misannotation and supports more comprehensive gene family identification. Moreover, existing research mainly focused on *StnsLTP* expression during tissue development, while systematic characterization of drought-responsive traits and drought-tolerance-related candidate genes remains limited. In this study, *StnsLTP* family members were re-identified from the potato DM6.1 genome using both PF00234 and PF14368 conserved domains, followed by comprehensive bioinformatic analysis. Three PEG-responsive candidate genes were screened from transcriptome data, and their expression patterns were validated by RT-qPCR under different drought treatment durations and in different potato tissues. This study improves the annotation completeness of the potato *nsLTP* gene family and systematically characterizes *StnsLTP* expression under drought stress. Our results demonstrate the potential roles of *StnsLTPs* in drought responses and provide valuable candidate gene resources for exploring drought-response mechanisms and future functional assays in potato.

## 2. Materials and Methods

### 2.1. Test Materials

In this study, the virus-free test-tube plantlets of the main potato cultivars ‘Kexin 1’, ‘Jinshu 16’ and ‘Jizhangshu 12’ in Inner Mongolia were used as experimental materials. ‘Kexin 1’ was bred by sexual hybridization with ‘347-128’ as the female parent and ‘Epoka’ as the male parent in 1958. The tuber is oval, the periderm is light yellow and smooth, the flesh is light yellow, and the number of eyes is small and the eyes are shallow. ‘Jinshu 16’ was bred by crossing ‘*NL 94014*′ as the female parent and ‘9333-11’ as the male parent. The tuber is round, the periderm is light yellow and smooth, the flesh is white, the eyes are deep and medium in size, and the tubers are set intensively with uniform shape and size. ‘Jizhangshu 12’ was bred by crossing ‘Atlantic’ as the female parent and ‘99-6-36’ as the male parent. The tuber is oblong, the periderm is light yellow and smooth, the flesh is white, the eyes are shallow, stolons are short, and tubers are packed. These cultivars have the characteristics of good commodity value, strong drought tolerance and wide adaptability. The experimental materials were provided by Ulanqab Academy of Agricultural and Forestry Sciences.

### 2.2. Drought Stress Treatment

The virus-free plantlets of ‘Kexin 1’, ‘Jinshu 16’ and ‘Jizhangshu 12’ were grown on Murashige and Skoog (MS) medium (Shanghai Macklin Biochemical Co., Ltd., Shanghai, China) and cultured in a constant temperature and humidity light incubator (Jiangsu Shenglan Instrument Manufacturing Co., Ltd., Yancheng, China) for 20–25 d. The temperature was 25 °C, the light intensity was 2000 lx, the photoperiod was a 16 h light/8 h dark. To determine the appropriate drought stress concentration, a preliminary experiment was conducted with 8 PEG 6000 (Dalian Meilun Biotechnology Co., Ltd., Dalian, China) concentration gradients (5%, 10%, 15%, 20%, 25%, 30%, 35%, 40%, *w*/*v*). Preliminary concentration screening was performed using test-tube plantlets of the three cultivars. Plantlets with uniform growth status were selected. The medium attached to roots was gently rinsed off with careful manipulation to avoid root damage. The plantlets were transferred into stress containers, and different PEG 6000 solutions were prepared with liquid MS medium and added to fully submerge the root system while keeping shoots above the liquid surface. Morphological changes were visually assessed. Concentrations causing rapid lethal wilting or showing negligible phenotypic changes were excluded. The 20% (*w*/*v*) PEG 6000 solution induced clear drought symptoms in potato plantlets without rapid lethal wilting and was therefore selected for simulated drought treatment. This concentration has been widely adopted in published potato drought stress studies [37]. Healthy and uniformly grown potato test-tube seedlings were subjected to simulated drought stress using the optimal 20% (*w*/*v*) PEG 6000 solution screened out in the preliminary experiment. Parallel control plantlets were immersed in PEG-free liquid MS medium under identical culture conditions. Control and treated samples were harvested at the same corresponding time points (0 h, 8 h, 12 h, 16 h, and 24 h). Each biological replicate contained six uniformly grown plantlets. After the stress treatment, the root, stem and leaf tissues of each variety of plants were collected at 0 h, 8 h, 12 h, 16 h and 24 h, respectively. The samples were immediately put into liquid nitrogen for quick freezing to inhibit enzyme activity and prevent nucleic acid degradation. Three biological replicates were established for both the treatment and control groups. The treated samples were stored in a −80 °C ultra-low temperature freezer for subsequent test analysis.

### 2.3. Selection of Candidate Genes for RT-qPCR Validation

RNA-seq expression data containing NaCl, ABA, IAA, GA3, BAP and heat treatments were downloaded from Spud DB (https://spuddb.uga.edu, accessed on 10 August 2025). ABA acts as a key signaling molecule in plant drought responses. Therefore, FPKM values of ABA-treated samples were extracted for preliminary screening to obtain candidate genes with high expression under ABA treatment, and *StnsLTP.115*, *StnsLTP.117* and *StnsLTP.118* were selected from these candidates for subsequent RT-qPCR validation.

### 2.4. Real-Time Fluorescence Quantitative (RT-qPCR) Analysis

The total RNA of the test samples was extracted by TRIZOL plant total RNA extraction kit (Invitrogen, Carlsbad, CA, USA). The purified total RNA was used as a template, and HiFi Script cDNA first-strand synthesis kit (Kangwei Century Biotechnology Co., Ltd., Taizhou, China; product number: CW2569) for cDNA reverse transcription synthesis. RT-qPCR amplification system used 2 × Real Star Fast SYBR qPCR Mix (Kangrun Biotechnology Co., Ltd., Shanghai, China; shipment No.:A301). The RT-qPCR specific primers were designed by Primer Premier 5.0 software, and the specific nucleotide sequence is shown in Table 1. The potato *Actin* gene was used as the internal reference gene to correct the relative expression of the target gene. Three biological replicates were set for all RT-qPCR reactions, and the relative gene expression was calculated and statistically analyzed by the 2^−ΔΔCt^ method [38].

### 2.5. Identification of StnsLTP Gene Family Members in Potato

In order to identify the *nsLTP* genes in potato, the genomic data of potato DM6.1 version was obtained from Spub DB database (https://spuddb.uga.edu/, accessed on 10 August 2025), and the protein sequence of tomato *nsLTP* gene was obtained from Phytozome13 database (https://phytozome-next.jgi.doe.gov/, accessed on 6 November 2025). In this study, Blastp and hidden Markov model (HMM) were combined for gene identification. The specific process is as follows: BlastP alignment was performed using the Blastcompare Two seqs [sets] function of TBtools-II (version v2.121) [39] software with the tomato nsLTP protein sequence as the reference sequence. The parameters were set to E-value = 1 × 10^−5^, Numof Threads = 2, Numof Hits = 500 [39]. Download the *nsLTP* family’s PF00234 and PF14368 HMM files from the pfam database (https://www.ebi.ac.uk/interpro/, accessed on 8 November 2025), and use the Simple HMM Search function of TBtools-II software to perform HMM comparison analysis. The identification results of the above two methods were integrated and submitted to the NCBI Conserved Domain Database (NCBI-CDD) (https://www.ncbi.nlm.nih.gov/Structure/cdd/cdd.shtml, accessed on 10 November 2025) for conservative domain verification, and the genes containing the complete nsLTP domain were screened as candidate members of the potato *StnsLTP* gene family [40].

### 2.6. Physicochemical Properties, Subcellular Localization and Transmembrane Domain Analysis of Potato StnsLTP Protein

The molecular formula, amino acid length, isoelectric point, molecular weight, instability coefficient, aliphatic coefficient, hydrophilicity and hydrophobicity of potato StnsLTP protein were analyzed by using the ProtParam tool of the Expasy database (https://www.expasy.org/, accessed on 12 November 2025) [41]. Subcellular localization and transmembrane domain prediction of StnsLTP protein were performed using Cell-PLoc 2.0 (http://www.csbio.sjtu.edu.cn/bioinf/Cell-PLoc-2/, accessed on 12 November 2025) and TMHMM 2.0 online tools (https://services.healthtech.dtu.dk/services/TMHMM-2.0/, accessed on 13 November 2025) [42,43].

### 2.7. Analysis of Cis-Acting Elements and Transcription Factors of Potato StnsLTP Gene Promoter

Using the Gtf/Gff3 Sequences Extract function of TBtools-II software, the 2000 bp sequence upstream of the start codon of each *StnsLTP* gene was extracted as the promoter region. Prediction and annotation of cis-acting elements using PlantCARE online tool (https://bioinformatics.psb.ugent.be/webtools/plantcare/html/, accessed on 17 November 2025); based on the results of PlantCARE analysis, the distribution characteristics of cis-acting elements were statistically and visually analyzed by TBtools-II’ Simple Bio Sequence Viewer and HeatMap functions [44]. The above promoter sequences were submitted to the PlantTFDB 5.0 database (https://planttfdb.gao-lab.org/, accessed on 19 November 2025) for systematic identification and analysis of transcription factors (TFs) potentially bound to the promoter region of the *StnsLTP* gene [45].

### 2.8. Construction of Phylogenetic Tree of Potato StnsLTP Gene Family

The reference genome data of tobacco and eggplant (*Solanum melongena*) were retrieved from the Sol Genomics Network database (https://solgenomics.net/, accessed on 21 November 2025). According to the identification process of potato *StnsLTP* gene in this study, the systematic identification of *nsLTP* gene family members in tobacco and eggplant was completed. Combined with the nsLTP protein sequences of potato, tomato, tobacco and eggplant identified in this study, the phylogenetic tree of the *nsLTP* gene family of the above four Solanaceae crops was constructed by using MEGA (version 11) software and the Maximum Likelihood (ML) method. Bootstrap repeated sampling verification times were set to 1000 times to evaluate the confidence of the phylogenetic tree branch. After exporting the constructed phylogenetic tree file to Newick format, it was uploaded to the iTOL online visualization platform (https://itol.embl.de/, accessed on 23 November 2025) for the beautification and visualization optimization analysis of the phylogenetic tree.

### 2.9. Prediction of miRNA and Protein Secondary Structure of Potato StnsLTP Gene

The secondary structure of potato StnsLTP protein was predicted and analyzed by SOPMA online tool (https://npsa.lyon.inserm.fr/cgi-bin/npsa_automat.pl?page=/NPSA/npsa_sopma.html, accessed on 24 November 2025), and the composition characteristics of α-helix, β-sheet, extended chain and random coil were predicted [46]. The coding sequence (CDS) of potato *StnsLTP* gene was used as the target, and the microRNA (miRNA) molecules interacting with it were predicted by PsRNATarget online target prediction platform (https://www.zhaolab.org/psRNATarget/, accessed on 25 November 2025) [47].

### 2.10. Analysis of Conserved Motifs and Gene Structure of Potato StnsLTP Gene Family

The conserved motifs of potato StnsLTP family proteins were predicted and analyzed by MEME Suite online tool (https://meme-suite.org/meme/, accessed on 26 November 2025). The parameters were set as follows: the maximum predicted number of conserved motifs was 10, and the length of motifs ranged from 6 to 50 amino acids. Based on the gene annotation file of potato DM6.1 genome, the distribution characteristics of exons, introns and non-coding regions of *StnsLTP* gene were analyzed. According to the results of conserved motif prediction and gene structure analysis, the Gene Structure View (Advanced) function of TBtools software was used for joint visualization analysis [44,48].

### 2.11. Chromosome Mapping and Homology Analysis of StnsLTP Gene Family Members in Potato

Using the Gene Location Visualize from GTF/GFF function module of TBtools-II software, the chromosome physical localization and visualization of *StnsLTP* gene were carried out based on the potato reference genome annotation file [44]. In order to analyze the collinearity distribution characteristics of *StnsLTP* gene in potato, this study used MCScanX (version 0.8) analysis tool to systematically identify the collinearity gene pairing relationship within the species of this gene family, and through the Advanced Circos function of TBtools-II software, the identified collinearity association information was converted into a Circos map to realize the intuitive visualization of gene collinearity relationship [49]. In order to clarify the evolutionary relationship between potato and *Arabidopsis thaliana*, rice, maize, tomato and tobacco *nsLTP* genes, the genome annotation files of *Arabidopsis thaliana*, rice and maize were obtained from the Ensembl Plants database (https://plants.ensembl.org/index.html, accessed on 27 November 2025), and the genome annotation files of tomato and tobacco were obtained from the Sol Genomics Network database. Through the One Step MCScanX function of TBtools-II software, the collinear relationship of *nsLTP* genes between different species was analyzed and visualized. The Simple Ka/Ks Calculator function module of TBtools-II software was used to quantitatively analyze the evolutionary selection pressure of potato *StnsLTP* gene family. The nonsynonymous substitution rate (Ka) and synonymous substitution rate (Ks) of gene sequences were calculated, and the Ka/Ks ratio was deduced to clarify the selection mode of the gene family in the evolutionary process [44].

### 2.12. GO Enrichment Analysis of Potato StnsLTP Gene Family

Using eggNOG-mapper (http://eggnog-mapper.embl.de/, accessed on 28 November 2025) to perform Gene Ontology (GO) functional annotation on the potato genome sequence. Subsequently, the GO annotation information of potato *StnsLTP* gene family was extracted from the annotation results by TBtools-II software, and the GO annotation information was visualized and analyzed by using the micro-bioinformatics platform (http://www.bioinformatics.com.cn, accessed on 29 November 2025).

## 3. Results

### 3.1. Identification, Physicochemical Properties and Subcellular Localization Analysis of StnsLTP Gene Family Members in Potato

Based on the conserved sequences of PF00234 and PF14368 domains, 123 potato *StnsLTP* gene family members were identified by BLASTP alignment and HMM search, and named as *StnsLTP.001*-*StnsLTP.123* according to chromosome localization (Supplementary Table S1). The analysis of the physical and chemical properties of the above StnsLTP proteins showed that the amino acid length was 84–390 amino acid residues (aa), and the molecular weight was 9.25–41.22 kDa. The coefficient of variation of these two parameters was about 35.5%, indicating that the family members had moderate differentiation in molecular size. Isoelectric point analysis revealed that the family was dominated by alkaline proteins (74.8%), with acidic and neutral proteins accounting for 21.1% and 4.1%, respectively. Hydrophobicity analysis showed that the members of the *nsLTP* family were obviously hydrophobic (average GRAVY value was 0.30), of which 91.1% were hydrophobic proteins, which was consistent with the lipid binding function of the *nsLTP* family. The results of protein stability showed that the family was dominated by unstable proteins, accounting for 56.9%, and the stability differences among members were obvious. The aliphatic index was relatively conserved among these proteins, suggesting a conserved structural feature of this family. Subcellular localization prediction showed that *StnsLTP* family members were mainly located in the cell membrane (47.2%) and cell wall (34.1%). A few were distributed among nine localization types, including the nucleus, cytoplasm, reflecting functional diversity. The transmembrane domain analysis showed that 56 (49.6%) members contained one transmembrane domain, only 6 (4.9%) members contained two transmembrane domains, and the rest had no transmembrane structure. Most proteins were predicted to possess transmembrane regions, which may support membrane association. The above results describe the basic sequence features and structural variation in potato *StnsLTP* family proteins.

### 3.2. Secondary Structure Analysis of Potato StnsLTP Protein

The secondary structure prediction results of potato StnsLTP family proteins (Supplementary Table S2) showed that the family proteins were composed of four conformations: α-helix, extended strand, β-turn and random coil. Among them, α-helix and random coil were the main structural components, which help maintain protein spatial conformation. The content of extended chain and β-turn is relatively low, and some members do not contain β-turn structure, suggesting that there are certain structural differentiation characteristics within the family. In general, the secondary structure of the family proteins is generally conserved, and the structural differences among the members may reflect structural variation within this family.

### 3.3. Motif and Gene Structure Analysis of Potato StnsLTP Gene

**Figure 1:**
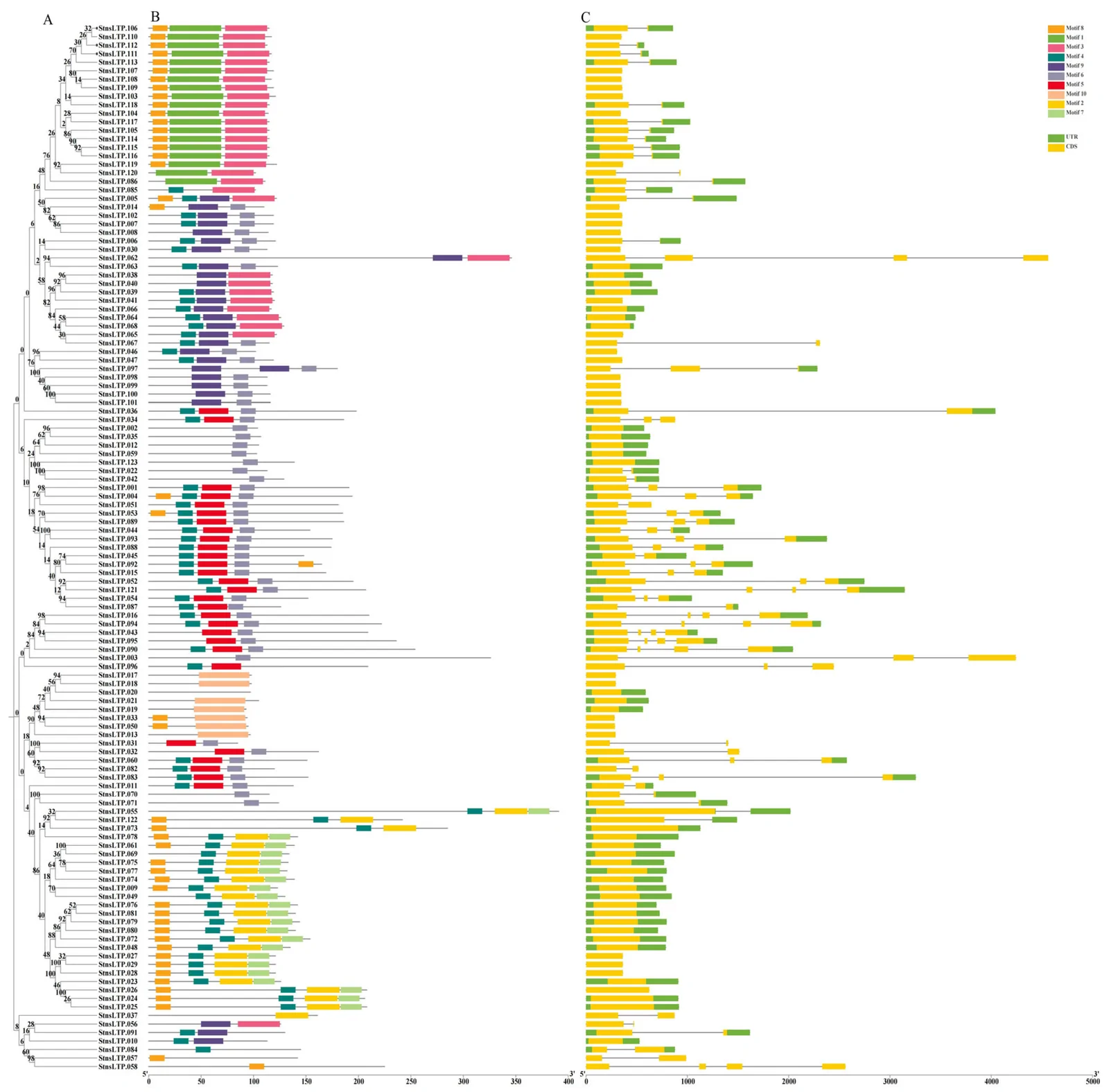
Evolutionary relationships, conserved motifs, and gene structures of potato *StnsLTP* family genes. (**A**) Evolutionary tree of StnsLTP proteins constructed using the Maximum likelihood (ML) method in MEGA 11, with 1000 bootstrap replicates. Bootstrap support values are labeled at branch node. (**B**) Conserved motifs of StnsLTP proteins identified by MEME. Each colored box represents a conserved motif (Motif 1-Motif 10, consistent with the figure legend), and different colors indicate different motifs. The scale bar at the bottom indicates the length of the motifs (amino acids). (**C**) Exon-intron structures of *StnsLTP* genes. Gray boxes represent exons (CDS), cyan boxes represent annotated untranslated region (UTR) fragments, and black lines represent introns. The scale bar at the bottom indicates the length of the genes (bp).

The conserved motifs of 123 *StnsLTP* genes were analyzed using the MEME tool, and a total of 10 conserved motifs were identified when MEME was configured to predict a maximum of 10 motifs (Figure 1A,B). The number of motifs in different genes ranged from 0 to 4, with most members containing 1–3 motifs. Among them, Motif 4 was the most widely distributed, present in 67 genes, suggesting it may serve as the core conserved motif of the family. Motif 10 was found in only seven genes, indicating it is a lineage-specific motif within this family. Each motif occurs only once per gene, with Motif 1 co-occurring with Motif 3 and Motif 2 co-occurring with Motif 7. Combined with the distribution characteristics of motifs, it is speculated that Motif 1 and Motif 7 may be derived motifs formed by the co-evolution of core motif 2 and motif 3, and their functions may be synergistic with the core motifs. The structural analysis of 123 *StnsLTP* family genes (Figure 1C) showed that there were some differences in the number of exons, introns and annotated untranslated region (UTR) fragments. The number of exons ranged from 1 to 4, and most of the genes contained one exon. The number of introns ranged from 0 to 4, and most members were concentrated in 0 to 1. The number of annotated UTR fragments ranged from 0 to 3. Most *StnsLTP* genes contained two annotated UTR fragments, and only *StnsLTP.055* had three annotated UTR fragments separated by introns. The above results showed that the gene structure of *StnsLTP* family members was generally similar, reflecting structural conservation across the family.

### 3.4. Evolutionary Relationship Analysis of Potato StnsLTP

**Figure 2:**
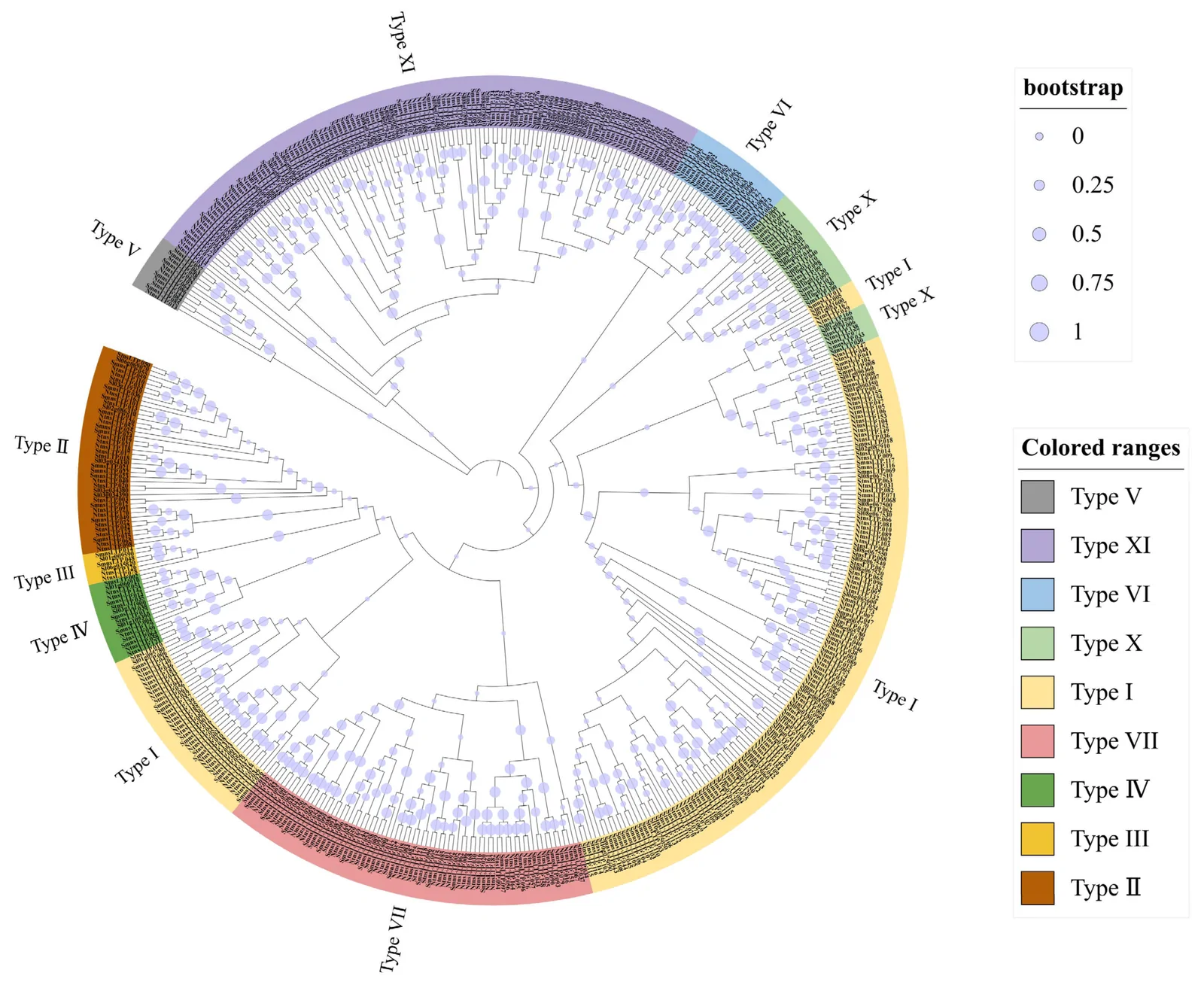
Evolutionary tree of nsLTP proteins from four Solanaceae crops. The tree includes 491 nsLTP proteins derived from potato (*Solanum tuberosum* L., St), tomato (*Solanum lycopersicum* L., Sl), tobacco (*Nicotiana tabacum* L., Nt) and eggplant (*Solanum melongena* L., Sm). The evolutionary tree was constructed using the Maximum likelihood (ML) method in MEGA 11 with 1000 bootstrap replicates, and the JTT+G amino acid substitution model, and was classified into nine distinct groups (Type I, Type II, Type III, Type IV, Type V, Type VI, Type VII, Type X and Type XI). Solid circles on branches represent bootstrap support values, and circle size is proportional to bootstrap values (range: 0–1). Different colors in the outer ring represent different evolutionary groups.

Referring to the published subfamily classification criteria of the tomato *nsLTP* gene family [31], the nsLTP protein sequences identified from potato, eggplant and tobacco in this study, together with the reported tomato nsLTP protein sequences, were used to construct a cross-species evolutionary tree of Solanaceae crops. The aim was to clarify the subfamily classification of the *nsLTP* gene family in Solanaceae species (Figure 2). A total of 123 *StnsLTP* genes in potato, 118 *SmnsLTP* genes in eggplant and 186 *NtnsLTP* genes in tobacco were identified in this study. Combined with 64 previously reported tomato *SlnsLTP* genes, a total of 491 protein sequences were analyzed. Multiple sequence alignment was performed on these protein sequences, and an evolutionary tree was constructed using the maximum likelihood (ML) method. Phylogenetic branching patterns were consistent with the classification rules of conserved nsLTP domains. Genes clustered within the same evolutionary clade exhibited close genetic relatedness, highly conserved protein sequences, and similar evolutionary divergence patterns. Based on evolutionary clustering analysis, the 491 *nsLTP* genes were classified into nine subfamilies with uneven member distribution across subgroups. Type III contained the fewest members, with only six genes accounting for 1.2% of the total, including two *SmnsLTP*, two *NtnsLTP* and two *SlnsLTP* genes; no potato *StnsLTP* members were detected in this subfamily. Type I was the largest subfamily, containing 179 genes and occupying 36.5% of the total number, among which there were 42 *StnsLTP*, 42 *SmnsLTP*, 71 *NtnsLTP* and 24 *SlnsLTP* genes. The remaining subfamilies differed obviously in gene quantity: Type Ⅺ contained 115 members, Type X 28, Type IV 16, Type II 41, Type V 11, Type VI 21, and Type VII 74. Subfamily composition varied greatly among different Solanaceae species. No tomato *nsLTP* members were identified in Type V, Type VI and Type VII, which may result from long-term evolutionary selection. Potato *StnsLTP* genes were distributed in all subfamilies except Type III with an uneven distribution pattern, and Type I had the largest member number of 42, accounting for 34% of all potato *nsLTP* genes. Furthermore, most potato *nsLTP* genes clustered closely with those of tomato and eggplant, indicating relatively close genetic relationships and reflecting high sequence conservation of homologous *nsLTP* genes among closely related Solanaceae species.

### 3.5. Chromosome Localization of Potato StnsLTP Gene

**Figure 3:**
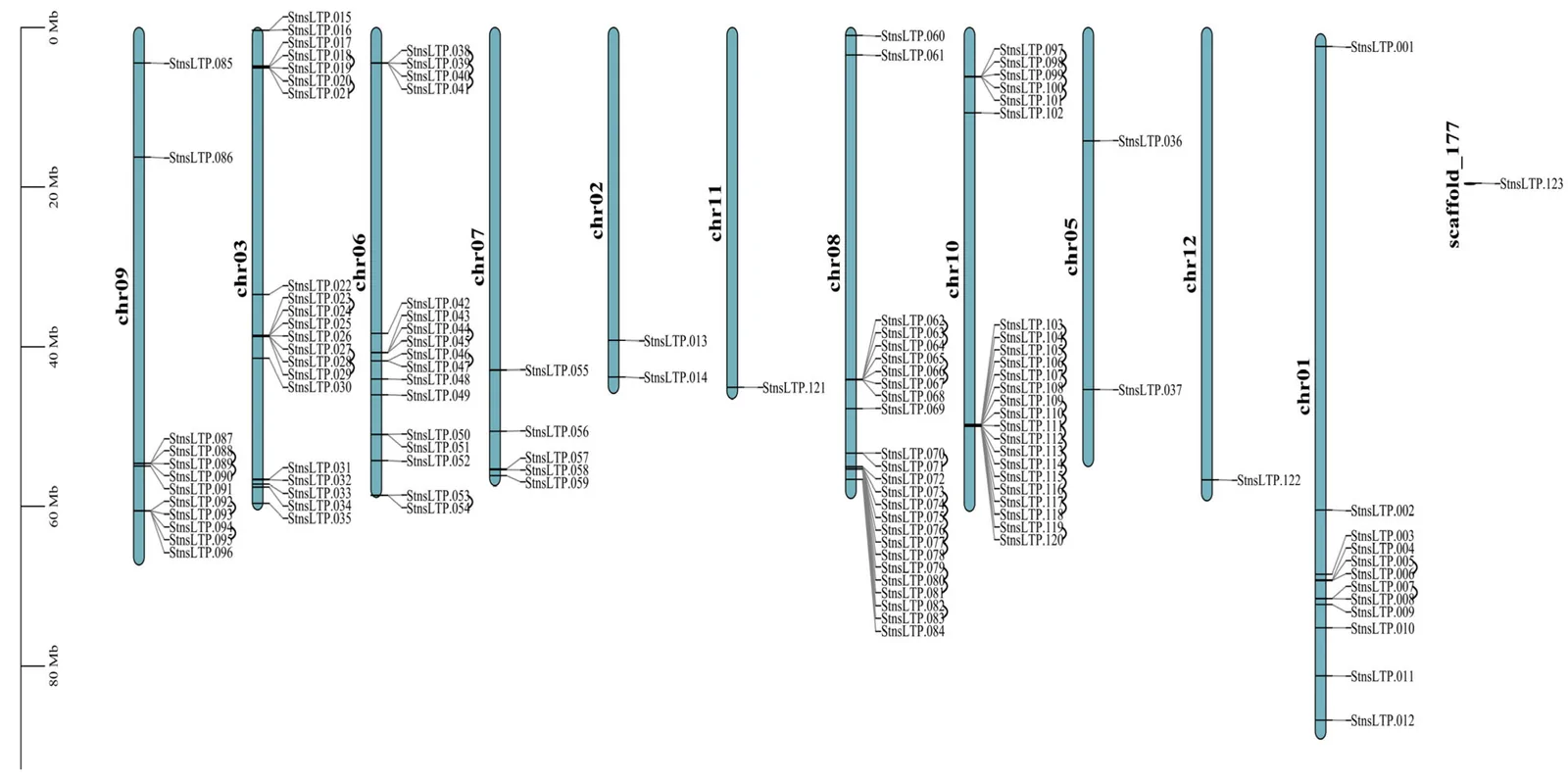
Chromosomal localization of potato *StnsLTP* genes. The left vertical scale represents the physical length of the potato chromosomes, with units in megabases (Mb). Among the 123 *StnsLTP* genes identified in this study, 122 genes were unevenly distributed across 11 potato chromosomes (chr01–chr03, chr05–chr09, chr10–chr12), and their physical positions are marked on the corresponding chromosomes. The remaining one gene (*StnsLTP.123*) was located on the unanchored scaffold_177.

The chromosome localization results of *StnsLTP* gene family in potato showed that 122 genes were unevenly distributed on 11 chromosomes among 123 members, which were chr01, chr02, chr03, chr05, chr06, chr07, chr08, chr09, chr10, chr11 and chr12, respectively. The remaining gene, *StnsLTP.123*, was anchored to unassembled scaffold_177. From the perspective of chromosome location distribution, *StnsLTP* genes on chr03, chr06, chr08 and chr09 are distributed in the middle and end of the chromosome, and the genes on the remaining chromosomes are mainly concentrated in the end. There were significant differences in the number of *StnsLTP* genes on each chromosome. Among these, Chromosome 1 contained 12 genes, including *StnsLTP.001* to *StnsLTP.012*; Chromosome 2 contained 2 genes, including *StnsLTP.013* to *StnsLTP.014*; Chromosome 3 contained 21 genes, including *StnsLTP.015* to *StnsLTP.035*; Chromosome 5 contained 2 genes, including *StnsLTP.036* to *StnsLTP.037*; Chromosome 6 contained 17 genes, including *StnsLTP.038* to *StnsLTP.054*; Chromosome 7 contained 5 genes, including *StnsLTP.055* to *StnsLTP.059*; Chromosome 8 contained 25 genes, including *StnsLTP.060* to *StnsLTP.084*; Chromosome 9 contained 12 genes, including *StnsLTP.085* to *StnsLTP.096*; Chromosome 10 contained 24 genes, including *StnsLTP.097* to *StnsLTP.120*; and only one gene was distributed on each of Chromosome 11 and Chromosome 12. Collectively, Chromosome 8 harbored the most *StnsLTP* genes, while Chromosomes 11 and 12 harbored the fewest (Figure 3 and Supplementary Table S3).

### 3.6. Analysis of Cis-Acting Elements of Potato StnsLTP Gene Promoter

**Figure 4:**
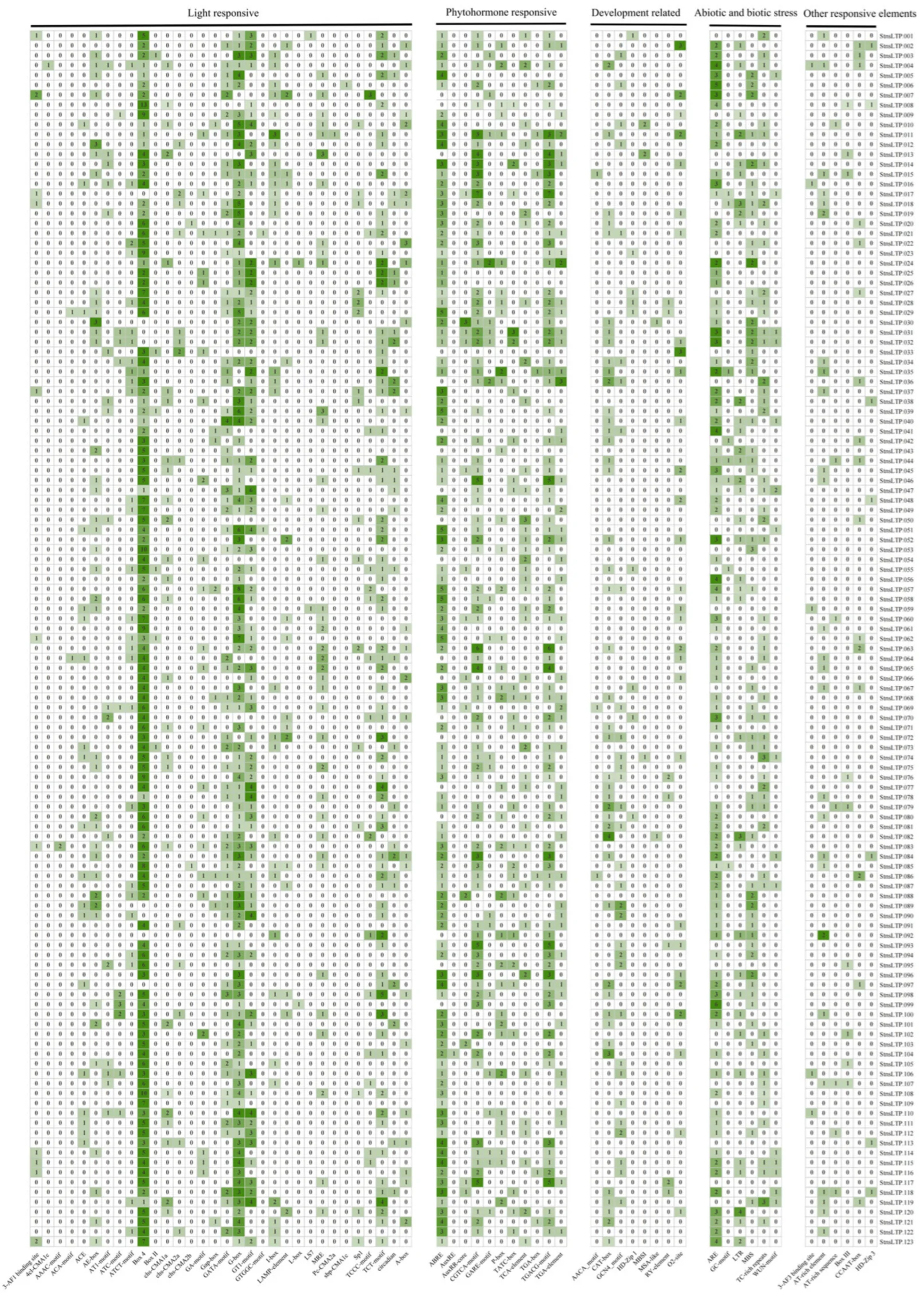
Distribution heatmap of cis-acting elements in the promoter regions of potato *StnsLTP* gene family. The figure shows the number and distribution of different cis-acting elements in the 2000 bp upstream promoter regions of each *StnsLTP* gene. All elements were classified into five categories based on their functions: light-responsive elements, hormone-responsive elements, growth and development-related elements, biotic and abiotic stress-responsive elements, and other regulatory elements. Each row represents an individual *StnsLTP* gene, and each column represents a specific cis-acting element. The color gradient (green intensity) indicates the number of corresponding cis-acting elements in each promoter.

Cis-acting elements within the 2000 bp upstream promoter region were systematically analyzed to investigate the potential regulatory mechanism of the potato *StnsLTP* gene family (Figure 4 and Supplementary Table S4). The results showed that after excluding CAAT-box, TATA-box and functional unannotated elements, a total of 63 cis-acting elements were identified and divided into five categories. Among them, 32 were light-responsive elements; 11 were hormone-responsive elements; 8 were elements related to growth and development; 6 were biotic and abiotic stress-responsive elements; and 6 belonged to other functional elements. The number of cis-acting elements of different *StnsLTP* genes was significantly different. *StnsLTP.038* and *StnsLTP.042* contained only 11 cis-acting elements, while *StnsLTP.119* and *StnsLTP.123* contained as many as 40.

The promoter region of potato *StnsLTP* gene contains a variety of stress and hormone response elements. Among them, the stress-related elements include drought response element MBS, low temperature response element LTR, mechanical damage and pathogen infection response element WUN-motif and hypoxia oxidative stress response element GC-motif. Hormone response elements include abscisic acid response element ABRE, auxin response element AUXRE/AUXRR-core, methyl jasmonate response element CGTCA-motif/TGACG-motif, etc. The presence of these motifs suggests that *StnsLTP* genes may possess potential binding sites for light, hormone and stress-related regulators.

### 3.7. Potential Transcription Factor Analysis of Potato StnsLTP Gene

**Figure 5:**
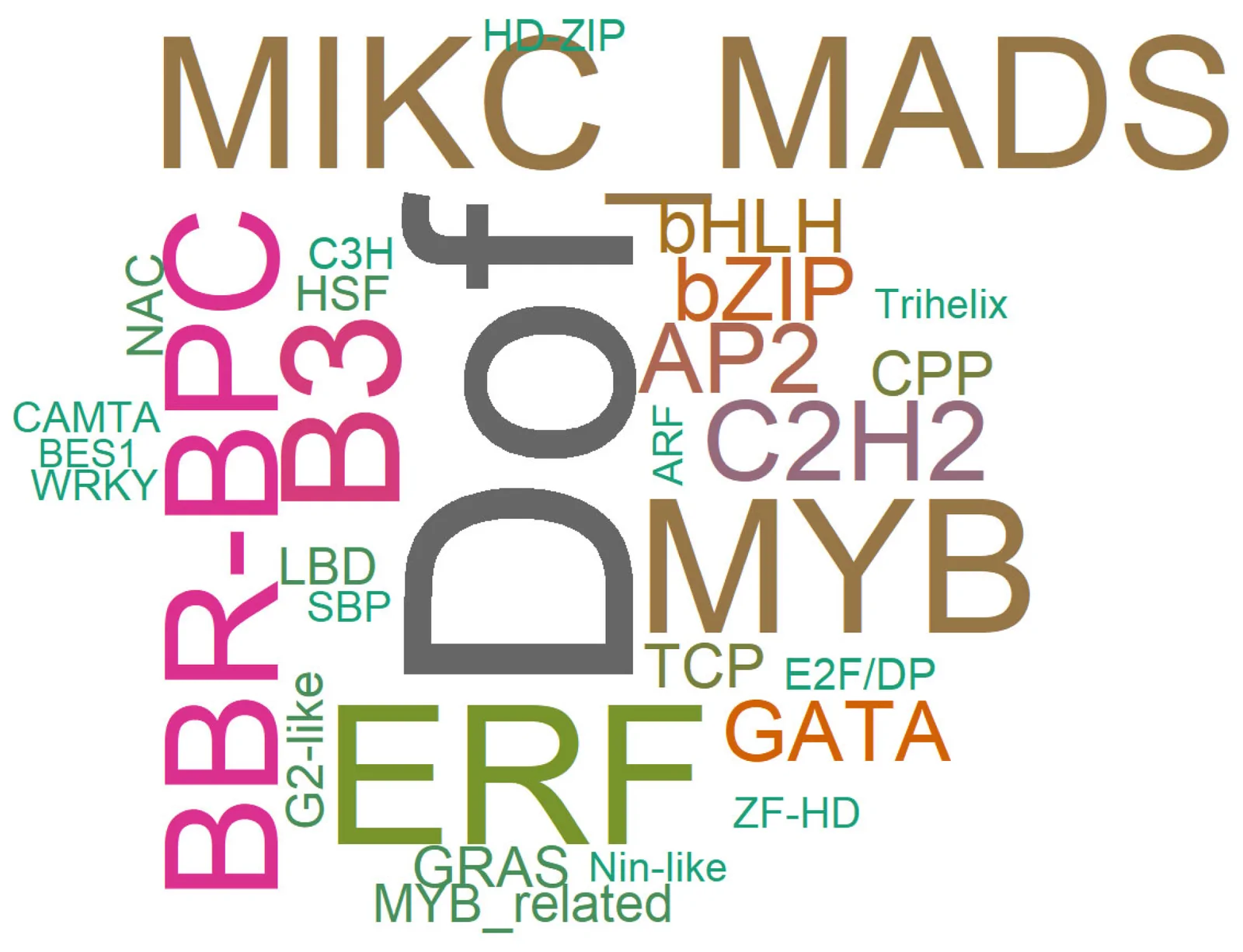
Word cloud of transcription factor (TF) binding sites in the promoter regions of potato *StnsLTP* genes. The word cloud visualizes the enrichment of predicted TF binding sites in the promoter regions of the potato *StnsLTP* gene family. The size of each word is positively correlated with the binding frequency of the corresponding TF family, where larger words represent higher enrichment frequency. Different colors in the word cloud are used for visual distinction and do not represent additional quantitative information.

TF binding sites were predicted to identify TFs that regulate *StnsLTP* expression. The analysis identified 30 candidate TFs potentially regulating *StnsLTP* genes (Figure 5). According to functional annotation, all identified TFs were classified into seven core functional categories (Figure 5): Growth regulation category (MYB, MYB-related, MIKC-MADS, TCP, LBD); Stress-response category (AP2, NAC, GRAS, HSF, WRKY); Hormone-signaling category (ARF, bZIP, B3, ERF, BES1); Metabolic-regulation category (Dof, C3H, C2H2, BBR-BPC, ZF-HD); Light/Temporal-response category (GATA, G2-like); Protein homeostasis and gene dynamics category (CPP, CAMTA, SBP); and General regulatory factor category (bHLH, HD-ZIP, E2F/DP, Nin-like). Among the TFs regulating 123 *StnsLTP* genes, Dof (194 bindings), ERF (107), MYB (102), MIKC-MADS (100), B3 (87), and BBR-BPC (82) stand out as high-frequency core TFs. These frequent predicted binding sites indicate that *StnsLTP* genes may contain potential binding regions for transcription factors involved in plant growth, stress response, metabolism and hormone signaling.

### 3.8. Construction of the Transcriptional Regulatory Network for the Potato StnsLTP Gene Family and Its Functional Analysis

A regulatory network was constructed between seven identified TF classes and their target *StnsLTP* genes in potato to characterize transcriptional regulatory patterns of this gene family. TFs from diverse functional categories displayed distinct regulatory preferences for *StnsLTP* family members, indicating cooperative transcriptional regulation via a complex multi-class TF network. Among stress-responsive TFs, AP2, WRKY, NAC, and others can target and regulate multiple *StnsLTP* genes, implying potential links between these TFs and *StnsLTP* sequences in stress-related regulatory modules (Supplementary Figure S1A). Among plant hormone signaling TFs, ARF, BZR1, ERF, and others likewise regulate multiple *StnsLTP* genes. Previous studies have shown that ARF, BZR1, and ERF participate in the signaling transduction of auxin, brassinosteroids, and ethylene [50,51,52], respectively, suggesting that *StnsLTP* genes may carry potential binding sites for these hormone-related TFs (Supplementary Figure S1B). In the category of growth-regulation TFs, MYB, MIKC-MADS, LBD, and others can regulate multiple *StnsLTP* members, implying potential associations between these TFs and *StnsLTP* sequences in growth-related regulatory modules (Supplementary Figure S1C). TFs involved in metabolic regulation, such as Dof, C2H2, BBR-BPC, and others, exhibit broad regulatory relationships with *StnsLTP* genes, indicating potential sequence-level associations between these TFs and the *StnsLTP* family members (Supplementary Figure S1D). TFs associated with light/temporal responses and protein homeostasis regulate only a subset of *StnsLTP* genes, indicating that regulatory patterns vary among *StnsLTP* genes (Supplementary Figure S1E,F). Shared TFs within this network may constitute core predicted regulatory modules associated with *StnsLTP* promoters (Supplementary Figure S1G). The above results provide a theoretical reference for elucidating the biological functions and stress response patterns of potato *StnsLTP* genes.

### 3.9. Evolutionary Characteristics, Collinearity, and Selection Pressure Analysis of Potato StnsLTP Genes

**Figure 6:**
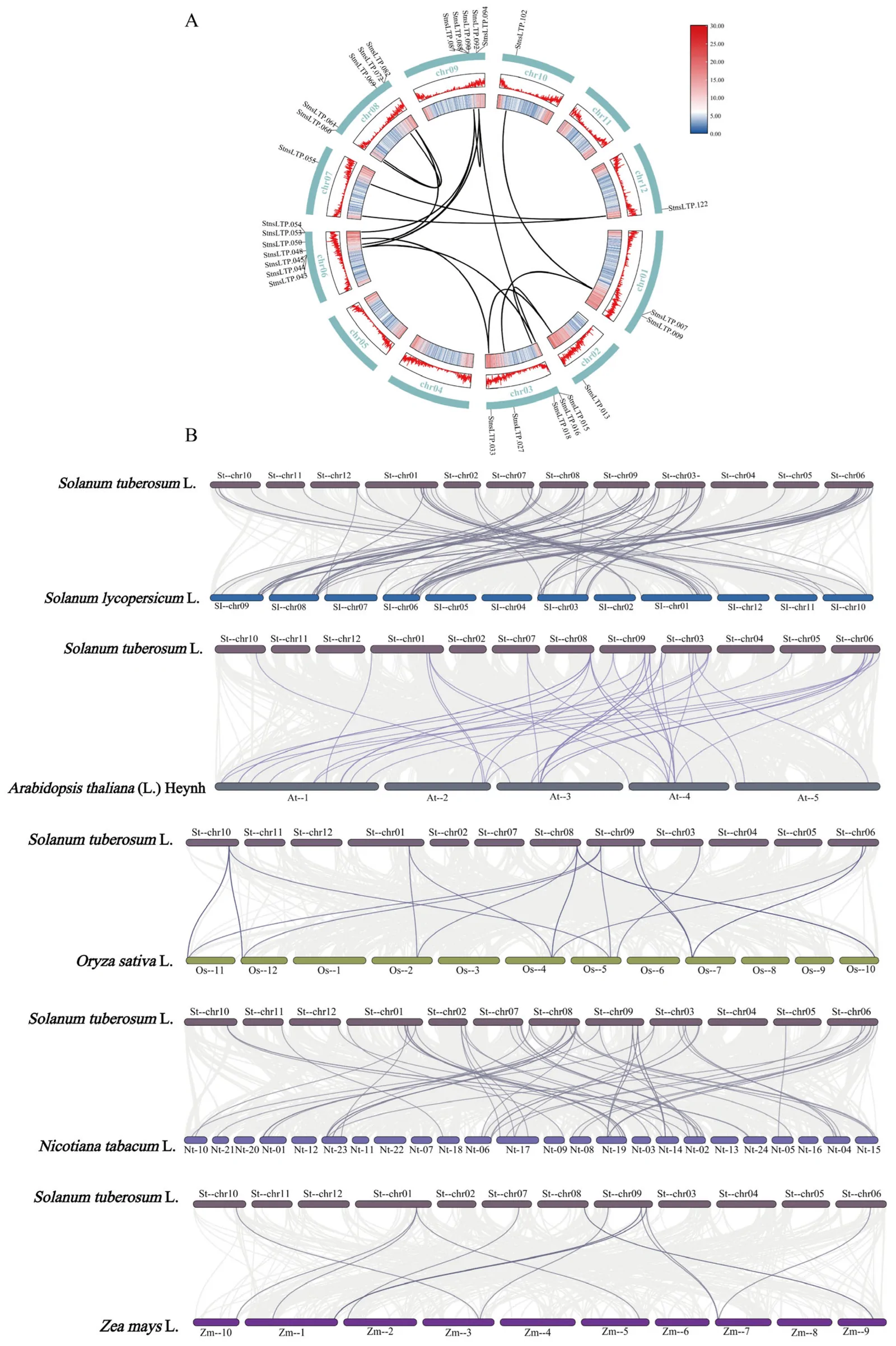
Intraspecific segmental duplication and interspecific collinearity analysis of the potato *StnsLTP* gene family. (**A**) Intraspecific segmental duplication analysis of *StnsLTP* genes in potato. The 12 potato chromosomes (chr01–chr12) are represented as blue arcs. Black lines inside the circle indicate segmental duplication relationships between *StnsLTP* gene pairs. The outer colored bar shows the gene density gradient across each chromosome. (**B**) Interspecific collinearity analysis of *nsLTP* genes between potato and five other plant species (tomato, *Arabidopsis*, rice, tobacco, and maize). Chromosomes of each species are represented as horizontal bars, labeled with species names and chromosome numbers. Purple lines highlight the collinear *nsLTP* gene pairs between potato and each species, while gray background lines represent the whole-genome collinearity.

To better understand the origin and functional divergence of the *StnsLTP* gene family, collinearity analysis of *StnsLTP* genes in potato was visualized using TBtools-II software (Figure 3 and Figure 6A). The results showed that a total of 45 tandem duplicate gene pairs and 20 segmental duplicate gene pairs were identified. The 20 segmental duplicated gene pairs were as follows: *StnsLTP.007*/*StnsLTP.102*, *StnsLTP.013*/*StnsLTP.018*, *StnsLTP.013*/*StnsLTP.033*, *StnsLTP.033*/*StnsLTP.050*, *StnsLTP.015*/*StnsLTP.045*, *StnsLTP.016*/*StnsLTP.043*, *StnsLTP.015*/*StnsLTP.092*, *StnsLTP.016*/*StnsLTP.094*, *StnsLTP.048*/*StnsLTP.072*, *StnsLTP.053*/*StnsLTP.088*, *StnsLTP.054*/*StnsLTP.087*, *StnsLTP.043*/*StnsLTP.094*, *StnsLTP.044*/*StnsLTP.092*, *StnsLTP.043*/*StnsLTP.090*, *StnsLTP.044*/*StnsLTP.088*, *StnsLTP.055*/*StnsLTP.122*, *StnsLTP.061*/*StnsLTP.069*, *StnsLTP.060*/*StnsLTP.082*, *StnsLTP.087*/*StnsLTP.092*, *StnsLTP.090*/*StnsLTP.094*. Among them, segmental duplication events were mainly concentrated on chromosomes 06 and 09, whereas tandem duplication events were more abundant on chromosomes 08 and 10. These results suggest that both tandem and segmental duplications contributed to the expansion of the *StnsLTP* family, and tandem duplication may have played a more prominent role in this process. Inter-species collinearity analysis (Figure 6B and Table 2) identified 104, 73, 45, 23 and 15 orthologous *nsLTP* gene pairs between potato and tomato, tobacco, *Arabidopsis*, rice and maize, respectively. The conservation of *nsLTP* genes was higher in dicots than in monocots, with the highest conservation observed among Solanaceae species. The closest collinear relationship was observed between potato and tomato, reflecting their close genetic relationship.

We further calculated Ka, Ks, and Ka/Ks ratios of duplicated gene pairs and interspecific orthologous gene pairs to evaluate evolutionary selection pressure of the *StnsLTP* gene family (Supplementary Table S5). For the 20 segmental duplication gene pairs, Ka/Ks values ranged from 0.1346 to 0.4509, while the 45 tandem duplication pairs ranged from 0.0748 to 4.0129. Most duplicated gene pairs had Ka/Ks < 1, indicating that the *StnsLTP* family was mainly subjected to purifying selection during evolution, with low amino acid substitution rates and relatively conserved protein sequences. The tandem duplication gene pair with the lowest Ka/Ks value (0.0748) showed strong purifying selection, and its low Ks value suggested a recent duplication event. By contrast, one tandem duplication pair had a Ka/Ks value of 4.0129 greater than 1, implying possible positive selection and sequence differentiation. Its extremely low Ks value indicated that this duplication event occurred recently.

In interspecific collinear analysis, the number of orthologous pairs was highest between potato and tomato (St-Sl, *n* = 102), followed by potato and tobacco (St-Nt, *n* = 71), potato and *Arabidopsis* (St-At, *n* = 27), and lowest between potato and rice (St-Os, *n* = 3). This pattern reflects the close phylogenetic relationship within Solanaceae (potato, tomato, tobacco), while the genetic relationship with *Arabidopsis* and the monocot rice was relatively distant. The Ka/Ks ratios of orthologous gene pairs between potato and other species ranged from 0.0438 to 2.1457, with most values below 1. This further indicated that purifying selection was the main evolutionary pattern of *nsLTP* genes. Only a small number of orthologous pairs had Ka/Ks > 1, suggesting that a few gene members may have experienced weak positive selection and sequence divergence during species separation.

### 3.10. MiRNA Analysis of Potato StnsLTP Gene

**Figure 7:**
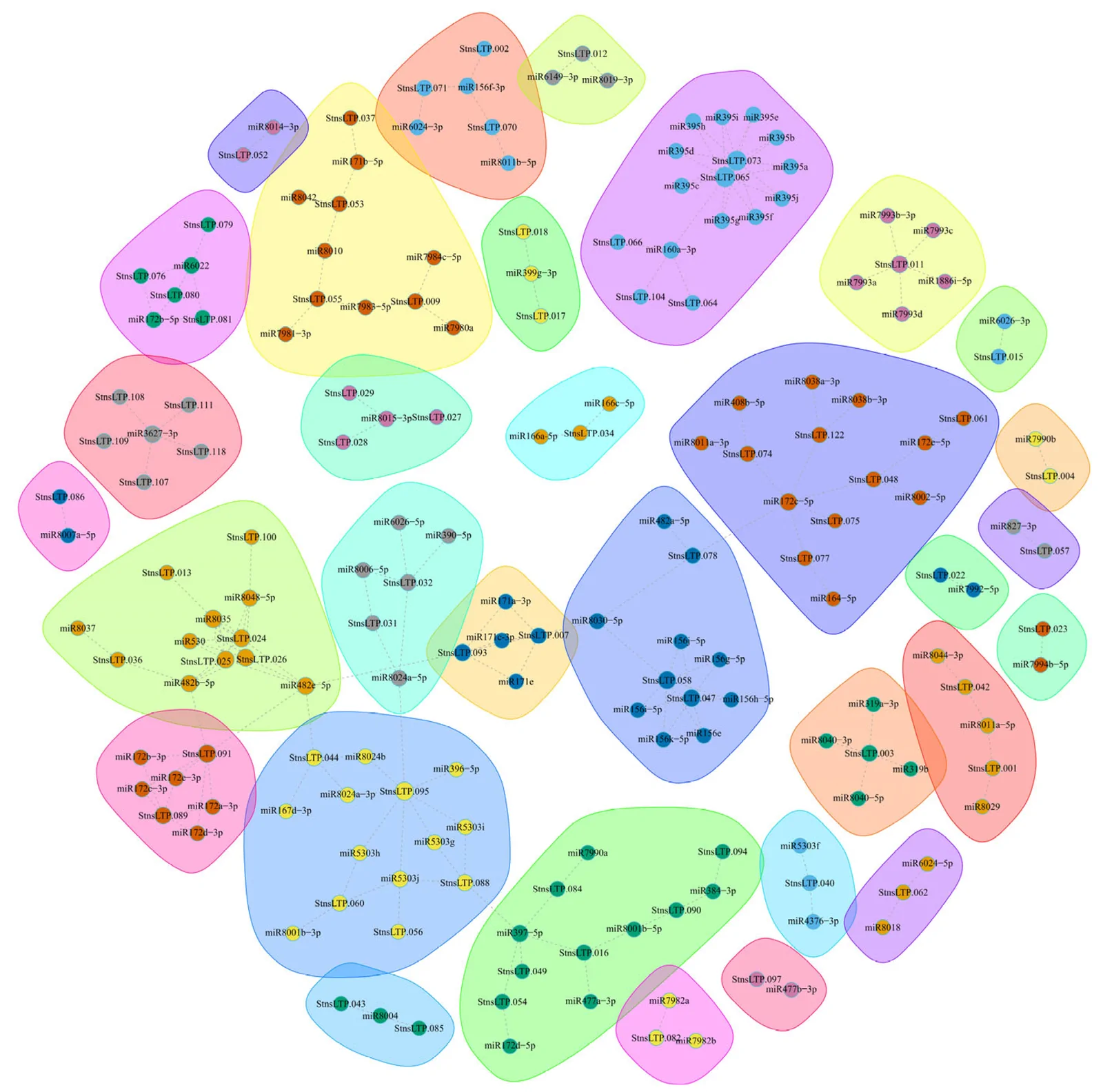
The miRNA-mRNA interaction network of potato *StnsLTP* genes. The network illustrates the predicted interactions between *StnsLTP* family genes and their target miRNAs. Circular nodes represent either *StnsLTP* genes or miRNAs, with different colors distinguishing interaction modules. The size of each *StnsLTP* gene node is positively correlated with the number of targeting miRNAs. Dotted lines indicate the potential regulatory interactions between miRNAs and their target *StnsLTP* genes.

Bioinformatics prediction analysis in this study revealed that *StnsLTP.002* and *StnsLTP.077* served as candidate target genes of miR156 and miR164, respectively. Meanwhile, *StnsLTP.107*, *StnsLTP.108*, *StnsLTP.109*, *StnsLTP.111* and *StnsLTP.118* were identified as potential target genes of miR3627 (Figure 7). This study preliminarily screened miRNAs with potential targeting relationships to potato *nsLTP* genes, providing candidate targets for further elucidating the regulatory correlation between them. MiRNAs are a class of crucial endogenous non-coding RNAs in plants, which can regulate gene expression at the post-transcriptional level [53]. Previous studies have shown that miR156 and miR164 are widely involved in plant responses to drought and other abiotic stresses, while miR3627 can respond to high and low temperature stresses and participate in plant immunity and stress defense processes [54,55,56]. Combined with the target gene prediction results, multiple potato *nsLTP* genes exhibit potential targeting relationships with miR156, miR164 and miR3627. These predicted interactions suggest that these miRNAs might associate with *StnsLTP* genes, and this prediction requires further experimental validation.

### 3.11. Gene Ontology Functional Enrichment Analysis of Potato StnsLTP Gene Family

**Figure 8:**
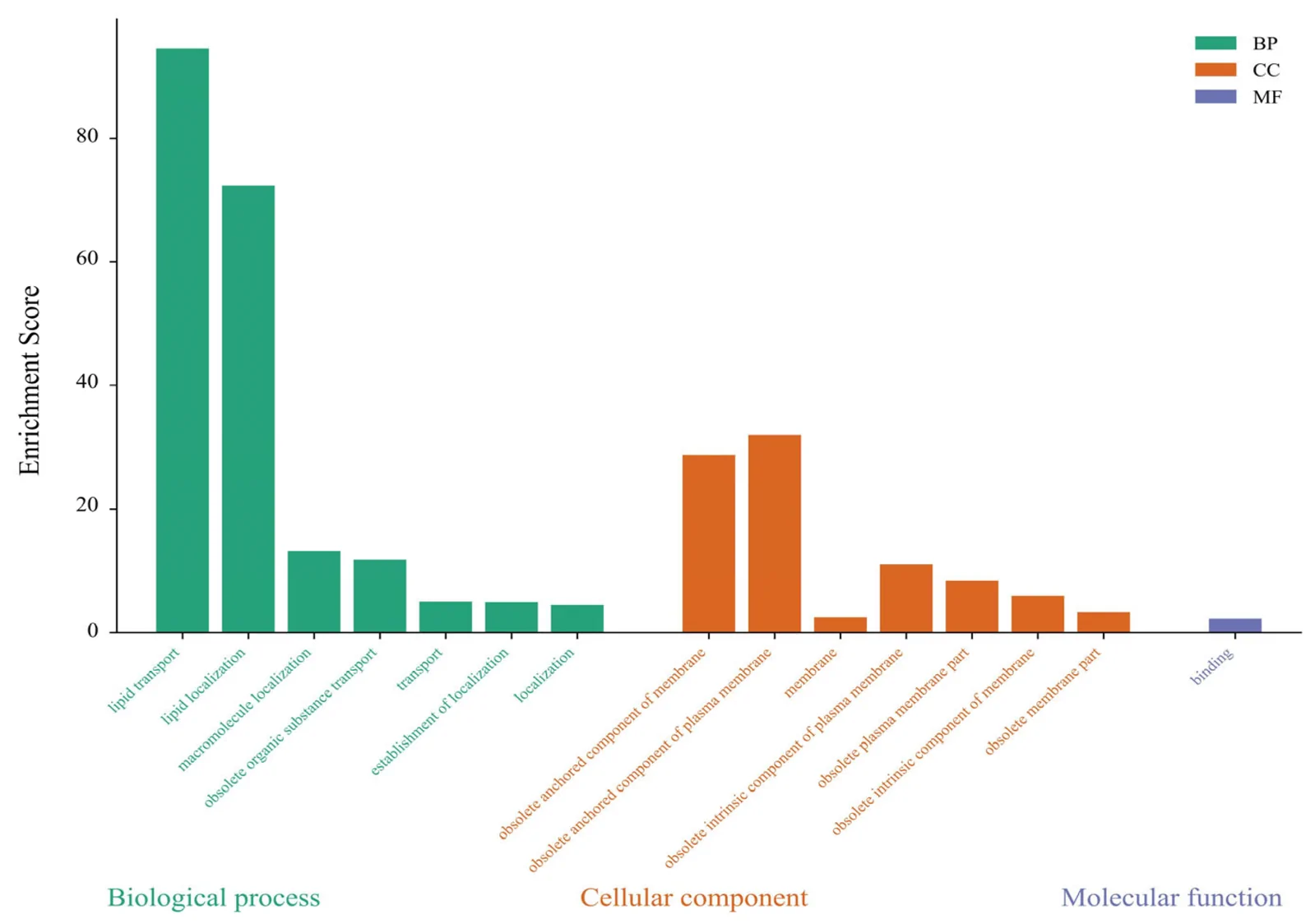
GO functional enrichment analysis of potato *StnsLTP* genes. The histogram shows the top enriched Gene Ontology (GO) terms for *StnsLTP* family genes, categorized into three main functional categories: biological processes (BP, green), Cellular Component (CC, orange), and Molecular Function (MF, purple). The vertical axis represents the enrichment score, which reflects the significance of enrichment for each GO term, with higher scores indicating more significant enrichment. The horizontal axis lists the names of the enriched GO terms.

Gene Ontology (GO) enrichment analysis was performed to investigate the potential biological functions of potato *StnsLTP* family genes (Figure 8 and Supplementary Table S6). *StnsLTP* family genes were mainly enriched in three functional categories, among which the enrichment signal of the biological process (BP) category was the most prominent. Lipid transport (GO:0008689) and Lipid localization (GO:0010876) showed extremely significant enrichment in biological processes (*p* < 0.01). Among the 16 GO-annotated genes in the *StnsLTP* family, 13 genes were annotated under these two entries, and the enrichment scores were as high as 94.50 and 72.38, respectively. This enrichment pattern suggests that *StnsLTP* genes are associated with lipid transport and lipid localization pathways. In addition, Macromolecule localization (GO:0033036), Obsolete organic substance transport (GO:0071702) and other items were also significantly enriched (*p* < 0.01). Although the enrichment degree was relatively weak, their functions were closely related to cell membrane stability and stress response, implying potential links between *StnsLTP* genes and stress-associated pathways. In the Cellular Component (CC) category, the *StnsLTP* genes were significantly enriched in Anchored component of plasma membrane (GO:0046658), Anchored component of membrane (GO:0031225), membrane (GO:0016020) and other functions (*p* < 0.01). Seventeen *StnsLTP* genes were annotated on the membrane. Although the number of genes annotated on the Anchored component of plasma membrane and Anchored component of membrane was small, the enrichment scores reached 32 and 28. These results indicate that StnsLTP proteins were mainly localized in the cell membrane system, which was highly consistent with its lipid transport function. The cell membrane is an important structural basis for plants to resist drought stress and maintain intracellular water balance, suggesting that membrane-localized StnsLTP proteins might be associated with membrane-related processes under stress. In the Molecular Function (MF) category, only binding (GO:0005488) showed extremely significant enrichment (*p* < 0.01), and no other significantly enriched functions were found. These findings indicate the functional characteristics of the *StnsLTP* family are mainly reflected in biological processes and cellular localization, and the specific molecular mechanism still needs further study.

These results indicated that the potato *StnsLTP* gene family is mainly involved in lipid transport and lipid localization processes, and its encoded proteins are primarily localized to the cell membrane system, which is consistent with the functional characteristics of plant non-specific lipid transfer proteins [7]. Previous studies have confirmed that lipid transport and membrane localization are closely associated with plant cuticular wax synthesis, membrane lipid remodeling, and the maintenance of cell membrane stability under stress conditions [57,58]. As an essential barrier for plants against drought stress, the cell membrane participates in drought responses by maintaining intracellular water balance, regulating reactive oxygen species (ROS) scavenging and osmotic adjustment. Combined with published knowledge, these GO annotations imply that *StnsLTP* genes might participate in lipid and membrane-associated pathways that are relevant to plant drought responses. Further analysis of gene expression patterns un der drought stress will help verify the functional association of this gene with drought resistance.

### 3.12. Expression Pattern Analysis of Potato StnsLTP Gene Under Drought Stress

**Figure 9:**
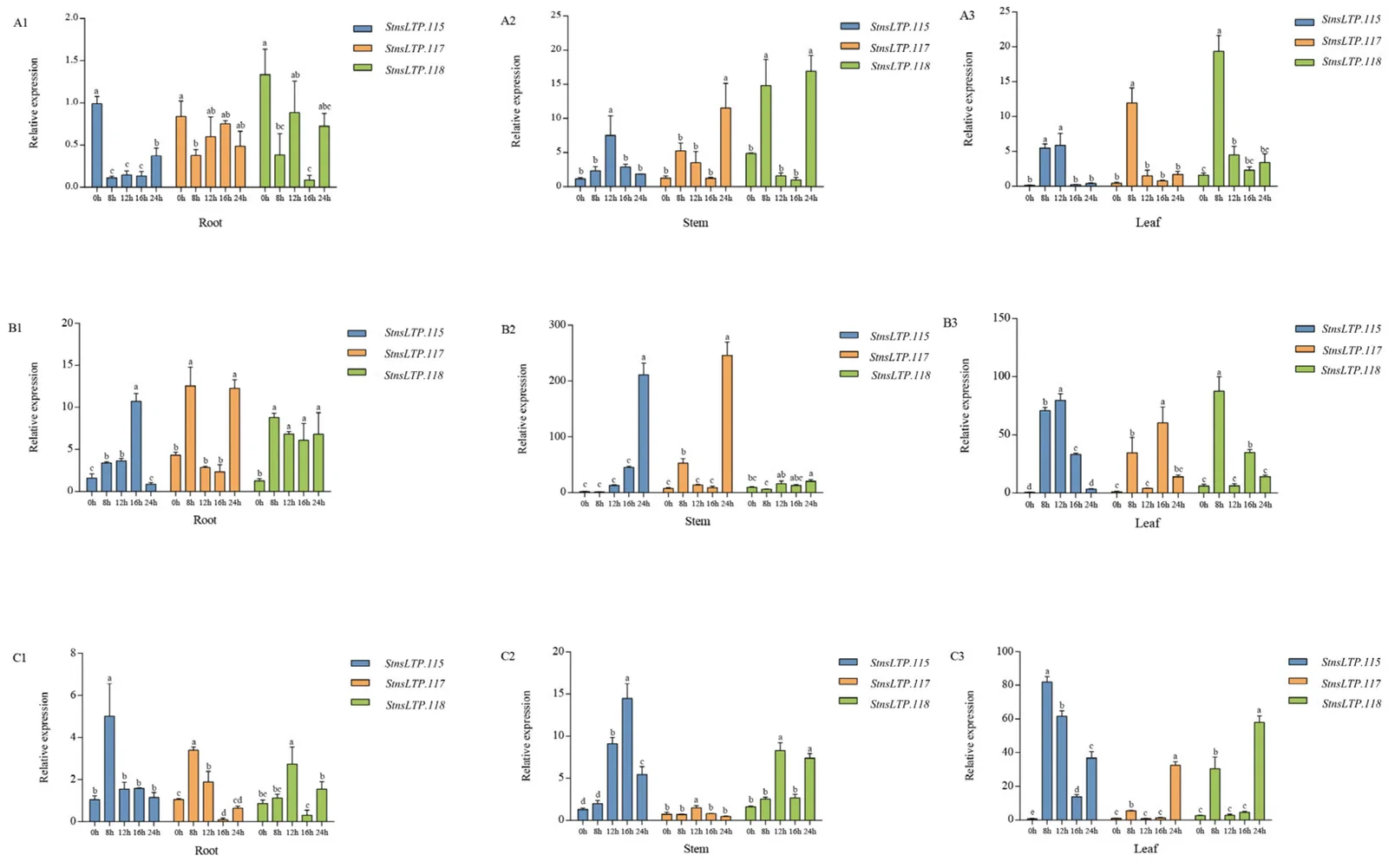
Expression patterns of *StnsLTP* genes in three potato cultivar under 20% PEG 6000-simulated drought stress. Panels (**A1**–**C3**) correspond to three potato cultivar, namely ‘Kexin 1’, ‘Jizhangshu 12’ and ‘Jinshu 16’. For each variety, the gene expression levels in three tissues including roots (**A1**,**B1**,**C1**), stems (**A2**,**B2**,**C2**) and leaves (**A3**,**B3**,**C3**) were displayed. Columns with different colors represent three genes of *StnsLTP.115*, *StnsLTP.117* and *StnsLTP.118*, respectively. The *x*-axis represents drought treatment time (0, 8, 12, 16, 24 h), and the *y*-axis represents the relative gene expression level. Different lowercase letters above the columns indicate significant differences (*p* < 0.05) in the expression levels of the same gene in the same tissue at different treatment time points. One-way ANOVA combined with Tukey′s multiple comparison test was used for statistical analysis, and there was a significant difference between two time points without shared lowercase letters.

Three genes (*StnsLTP.115*, *StnsLTP.117* and *StnsLTP.118*) with high expression and significant upregulation in drought-related RNA-seq data from the Spud DB potato genome database were selected for further validation to characterize the expression characteristics of *StnsLTP* genes under drought stress. Drought stress was simulated using 20% PEG 6000, and the expression patterns of these three genes in roots, stems, and leaves of virus-free seedlings of three potato cultivars ‘Kexin 1’, ‘Jizhangshu 12’, and ‘Jinshu 16’ were explored. The results showed that the expression patterns of *StnsLTP.115*, *StnsLTP.117*, and *StnsLTP.118* were significantly different in different potato cultivars, different organs, and different stress time points (Figure 9).

In the roots of ‘Kexin 1’, the three *StnsLTP* genes had higher basal expression at the initial stage of stress (0 h). As the stress time increased, the overall expression level was down-regulated. *StnsLTP.115* rapidly down-regulated and maintained a low level after stress. *StnsLTP.117* was significantly down-regulated at 8 h and then gradually increased. *StnsLTP.118* showed two up-regulated peaks at 12 h and 24 h, showing the characteristics of fluctuating expression (Figure 9(A1)). In the stem, the expression of the three *StnsLTP* genes showed an overall upward trend. The expression of *StnsLTP.115* and *StnsLTP.118* increased first and then decreased, and reached the peak at 12 h and 24 h, respectively. *StnsLTP.117* was continuously up-regulated and reached the highest expression level at 24 h (Figure 9(A2)). In leaves, the three *StnsLTP* genes showed an expression pattern of increasing first and then decreasing. Among them, the expression of *StnsLTP.115* peaked at 8 h and 12 h, while the expression of *StnsLTP.117* and *StnsLTP.118* peaked at 8 h (Figure 9(A3)).

In the roots of ‘Jizhangshu 12’, the expression of three *StnsLTP* genes was induced by drought stress, and the overall trend was up-regulated. The expression level of *StnsLTP.115* increased first and then decreased, and reached the highest at 8 h. The expression of *StnsLTP.117* was the highest at 8 h and 24 h. The expression level of *StnsLTP.118* was the lowest at 0 h, and it was significantly up-regulated at 8 h and maintained at a high expression level (Figure 9(B1)). In the stem, the expression of three *StnsLTP* genes was significantly different. The expression levels of *StnsLTP.115* and *StnsLTP.117* were the highest at 24 h. In contrast, *StnsLTP.118* was maintained at a low expression level throughout the stress cycle (Figure 9(B2)). In leaves, the three *StnsLTP* genes showed a trend of increasing first and then decreasing. The expression peak of *StnsLTP.115* appeared at 8 h. *StnsLTP.117* peaked at 12 h; *stnsLTP.118* had the highest expression level at 16 h (Figure 9(B3)).

In the roots of ‘Jinshu 16’, the expression levels of *StnsLTP.115* and *StnsLTP.117* increased first and then decreased, and both reached the highest at 8 h, while *StnsLTP.118* reached the peak at 12 h, and the expression level at 24 h was not significantly different from that at 0 h and 8 h (Figure 9(C1)). In stems, the expression level of *StnsLTP.115* peaked at 16 h and then decreased. The expression level of *StnsLTP.117* was the highest at 12 h. The expression of *StnsLTP.118* at 12 h and 24 h was significantly higher than that at 0 h, 8 h and 16 h (Figure 9(C2)). In leaves, the expression levels of the three *StnsLTP* genes showed an overall ‘up-down-up’ expression pattern. The expression level of *StnsLTP.115* reached the highest at 8 h. The expression levels of *StnsLTP.117* and *StnsLTP.118* showed the first peak at 8 h, then decreased, and reached the highest level at 24 h (Figure 9(C3)).

In summary, under drought stress, the expression patterns of *StnsLTP.115*, *StnsLTP.117* and *StnsLTP.118* in different potato cultivars and different organs were significantly different, suggesting these three genes display distinct expression responses to PEG-simulated drought among different potato cultivars and organs.

## 4. Discussion

### 4.1. Identification and Evolutionary Characteristic Analysis of the nsLTP Gene Family

Abiotic stress is one of the important factors limiting global agricultural production [59]. *nsLTPs* are encoded by multiple gene families. The number of *nsLTPs* family members varies significantly among different species and plays an important role in plant growth and development and stress response [60]. Previous studies have shown that 64,246 and 136 *nsLTP* genes were identified in the genomes of tomato [31], *Brassica napus* [24] and cotton (*Gossypium hirsutum*) [35], respectively, illustrating substantial interspecies variation in family size. Here, we identified 123 *StnsLTP* genes in the potato genome, all containing the canonical nsLTP conserved domain (Supplementary Table S1). The conservation and diversity of gene structure play a key role in the functional differentiation of gene families [61]. *StnsLTP* genes carry 1–4 exons and 0–4 introns with evident structural variation, and a large fraction are intronless. This feature agrees well with *nsLTP* families reported in other land plants [62]. Previous studies have shown that the *nsLTP* family originated from early differentiated terrestrial plants, and intron loss is an important event in the evolution of the family, which might partly explain the variation in intron numbers observed among *StnsLTP* genes in our study (Figure 1) [63].

To explore the evolutionary characteristics of the *nsLTP* gene family in Solanaceae crops, we performed genome-wide identification of *nsLTP* genes based on the updated potato DM6.1 chromosome-level reference genome, and identified 123, 118 and 186 *nsLTP* genes from the genomes of potato, eggplant and tobacco, respectively. An evolutionary tree was constructed using the identified *nsLTP* genes from potato, eggplant, tobacco, together with 64 known tomato *nsLTP* genes (Figure 2). Evolutionary analysis revealed that Solanaceae *nsLTP* family genes were clustered into nine evolutionary clades, with obvious differences in gene number among different clades (Figure 2). Notably, no *nsLTP* members of tomato were identified in subgroups V, VI and VII. The *StnsLTP* genes of potato were distributed unevenly in all subfamilies except subgroup III. Most potato *nsLTP* genes clustered closely with their homologous genes from tomato and eggplant in the evolutionary tree, indicating that the *nsLTP* homologous genes among closely related Solanaceae species possess close genetic relationships and high sequence conservation. Previous research using the potato DM v4.03 genome identified 83 *nsLTP* genes, which were grouped into eight subtypes, with no members assigned to types III, VI, IX or XI [64]. Comparison of gene loci between DM v4.03 and DM v6.1 annotations uncovered 61 shared genes (Supplementary Table S7). These differences may largely stem from revisions to the potato genome assembly and re-annotation of gene structures. The refined DM v6.1 assembly may improve gene prediction accuracy, facilitating the discovery of additional *nsLTP* family members in the present study. Additional published evidence indicated that type III, V, VI and VIII *nsLTP* genes are rarely distributed in Solanaceae, and type VII *nsLTP* genes are unique to monocots, which have not been identified in dicotyledonous potato [65]. The classification results in this study differed from the above findings. On the one hand, species-specific gene duplication and gene loss events occurred during the speciation and differentiation of Solanaceae species, resulting in divergent subtype distribution of the gene family [66]. On the other hand, the genome version 4.3 was constructed using short-read sequencing, which has fragmented contigs and incomplete annotation in repetitive and low-copy gene regions. In comparison, the DM6.1 genome adopted long-read and Hi-C assembly, providing substantially improved continuity, completeness, and accuracy of gene model prediction [67].

### 4.2. Chromosomal Distribution and Cis-Regulatory Element Profiling of Potato StnsLTP Genes

*nsLTP* genes exhibit non-random and uneven chromosomal distribution patterns, which are primarily driven by genome tandem duplication and segmental duplication events during evolution [68]. Consistent with this evolutionary pattern, 122 *StnsLTP* genes were unevenly mapped to 11 potato chromosomes, while *StnsLTP.123* lacked reliable chromosomal assignment (Figure 3). This indicates tandem and segmental duplications underpin the chromosomal distribution of this gene family. Although family members share high sequence homology, their biological functions have undergone remarkable functional divergence. Cis-acting elements in promoters modulate gene spatio-temporal expression patterns, and promoter analysis can reveal the transcriptional mechanisms responsible for functional differentiation among family genes [69].

Accumulating evidence demonstrates that *nsLTP* genes are widely involved in plant responses to drought, salt stress and pathogen infection [63]. Potato *StnsLTP* promoters harbor abundant cis-elements responsive to drought (MBS), low temperature (LTR), pathogen damage (WUN-motif), MeJA (CGTA-motif, TGACG-motif) and ABA (ABRE) (Figure 4), implying potential links between this gene family and diverse abiotic and biotic stress pathways. Abscisic acid and methyl jasmonate play vital roles in plant stress signal transduction [70,71], suggesting that *StnsLTP* genes might modulate stress-associated processes in potato through phytohormone signaling pathways. Many binding sites of bHLH, MYB, NAC and Dof TFs were also detected in the promoter sequences. These TFs modulate plant stress tolerance via ABA signaling pathways, which suggests that *StnsLTP* genes might participate in regulatory processes related to potato drought responses. Similar stress-and hormone-related elements have been detected in *nsLTP* promoters of barley (*Hordeum vulgare*) and *Brassica napus* [24,60], suggesting conserved stress-regulatory functions of the *nsLTP* family across plant species. In summary, *StnsLTP* genes may be coregulated by phytohormones and transcription factors, and could be potentially involved in potato growth, development, and responses to various abiotic and biotic stresses.

### 4.3. Duplication Expansion and Evolutionary Analysis of Potato StnsLTP Gene Family

Gene duplication events, including whole-genome duplication, segmental duplication and tandem duplication, drive the expansion and functional divergence of plant gene families [72]. Previous studies have pointed out that tandem repeats play a leading role in the expansion of the *nsLTP* family in cotton, barley. The tandem repeats of *nsLTP* gene family in cotton accounted for 47.82%, and 51.43% in barley [35,73]. Likewise, potato contains more tandem duplication pairs (45 pairs) than segmental duplication pairs (20 pairs) (Figure 6 and Supplementary Table S5). A greater number of tandem duplication events have also been documented in potato *nsLTPs* in previous comparative genomic studies, suggesting that tandem duplication acts as an important evolutionary mechanism driving *nsLTP* family expansion across diverse plant species [68]. Interspecific collinearity revealed abundant *nsLTP* homologous pairs between potato and other Solanaceae species, while only a small number of homologs were detected between potato and the monocot rice (Figure 6 and Supplementary Table S5). Potato and tomato belong to the same Solanaceae family and diverged relatively recently in evolution. Their genomes retain high synteny and a large number of conserved homologous loci, which explains the close genetic relationship observed between their *nsLTP* genes. By contrast, rice is a monocot species with an ancient evolutionary separation from Solanaceae, resulting in far fewer homologous gene pairs. These results revealed that potato shares a closer phylogenetic relationship with other Solanaceae species, and a relatively distant relationship with monocot rice, which is consistent with plant phylogeny [74].

Redundant copies generated by gene duplication can alter gene expression patterns through mutations in coding sequences and variations in cis-acting elements, thereby promoting functional differentiation [66,75]. Previous evolutionary analyses of rice and wheat *nsLTP* genes revealed that different subgroups of this family experienced distinct selective pressures. The core subgroup A with Ka/Ks < 1 underwent long-term purifying selection and maintained high sequence conservation, whereas other subgroups were mainly under positive selection and evolved at a faster rate. Although rice and wheat experienced independent gene duplication events after speciation, their homologous genes shared similar evolutionary selection patterns. Homologous genes under purifying selection still exhibited species-specific differences in stress response and tissue expression, indicating diverse differentiation patterns during long-term evolution. The Ka/Ks ratios of duplicated gene pairs and cross-species homologous genes in foxtail millet (*Setaria italica*) *nsLTP* family were all less than 1, indicating strong evolutionary constraints and limited functional differentiation derived from gene duplication events [76]. Comparative evolutionary analysis of Solanaceae *nsLTP* genes also revealed conserved evolutionary characteristics among species accompanied by lineage-specific differentiation [77]. Evolutionary selection pressure analysis in this study showed that the potato *nsLTP* gene family was generally under strong purifying selection, and most genes maintained highly conserved protein sequences. Tandem duplicated gene pairs displayed diverse evolutionary patterns; most were constrained by purifying selection, while a few pairs with Ka/Ks > 1 were subjected to positive selection. Cross-species homologous gene comparison revealed that only partial homologous genes between potato and tomato underwent positive selection, and purifying selection dominated among other species. This pattern arises because most *nsLTP* genes maintain essential biological functions and are restricted by strong purification selection, while a small fraction of duplicated genes accumulate mutations and undergo positive selection to adapt to species-specific environmental pressures. This indicates that the potato *nsLTP* family is evolutionarily conservative as a whole, with only a small number of genes showing species-specific differentiation among closely related species (Supplementary Table S5). The results of this study are consistent with previous reports on *nsLTP* families in Poaceae, foxtail millet and other Solanaceae, supporting the evolutionary pattern of overall conservation and local differentiation.

### 4.4. Expression Pattern of Potato StnsLTP Gene and Its Response to Drought Stress

Gene expression patterns can effectively reveal plant stress responses, growth and development regulation as well as tissue-specific expression characteristics, providing an important basis for subsequent gene function verification and mechanism analysis [78]. Studies have confirmed that *nsLTP* genes are widely involved in plant response and regulation to biotic and abiotic stresses. Sugarcane (*Saccharum officinarum*) *nsLTP* gene can positively regulate resistance to leaf burning. Among them, *ShnsLTP1.8*, *ShnsLTP1.10* and *ShnsLTPGb.1* are highly expressed in resistant cultivars and lowly expressed in susceptible varieties [79]. Barley *HvLTP2.1* and *HvLTP2.2* responded significantly to drought, low temperature and salt stress in roots [73]. In this study, based on transcriptome data, three PEG-responsive candidate *StnsLTP* genes, *StnsLTP.115*, *StnsLTP.117* and *StnsLTP.118*, were screened. These genes might be associated with drought stress responses. The expression of *StnsLTP.115*, *StnsLTP.117* and *StnsLTP.118* in three potato cultivars, ‘Kexin 1’, ‘Jizhangshu 12’ and ‘Jinshu 16’, was analyzed under PEG 6000 simulated drought stress. The expression levels of *StnsLTP.115*, *StnsLTP.117* and *StnsLTP.118* were significantly different in different cultivars and different tissues (Figure 9). This cultivar- and tissue-specific expression pattern indicates divergent expression responses to PEG-simulated drought across potato cultivars and tissue types.

Expression patterns of *nsLTP* genes differ among plant species in response to drought stress. In *Castanea mollissima*, *CmnsLTP6.9* peaked at 6 h of drought treatment and then decreased, showing an up-down pattern [27]. In *Beta vulgaris*, *BvM14-nsLTP* was induced in both leaves and roots with the same trend, reaching maximum expression at 6 h, and the induction fold in leaves was much higher than in roots, indicating obvious tissue specificity [80]. In rice, the drought-induced *nsLTP* gene *OsDIL* is strongly activated by drought, and its overexpression significantly enhances drought tolerance [81]. In tobacco, *NtLTP4* is rapidly induced by drought stress, with a 7.2-fold upregulation at 8 h, confirming its role in drought response [82]. Compared with previous studies, the three *StnsLTP* genes in potato exhibited more complex drought responses with strong cultivar-specific and tissue-specific expression patterns (Figure 9). In *Castanea mollissima* and sugar beet (*Beta vulgaris*), *nsLTP* genes showed consistent overall expression trends across tissues despite minor differences. In contrast, the three *StnsLTP* genes differed markedly in expression among the three potato cultivars. Their expression patterns varied substantially across the three tested potato cultivars, with distinct responses observed among roots, stems and leaves (Figure 9). Such cultivarspecificity may be related to variations in drought tolerance, genetic background, and stress regulatory mechanisms among potato cultivars. The tissue specificity of *StnsLTP* genes in potato is also more complex than that in sugar beet.

The response intensity and regulatory direction of *nsLTP* genes vary across species. In *Chimonanthus praecox*, *CpLTPI.1* was strongly induced by drought, whereas *CpLTPI.2* and *CpLTPI.3* were repressed, suggesting functional divergence among family members [83]. In this study, the three *StnsLTP* genes were predominantly upregulated under drought stress, but obvious downregulation was also observed in some tissues. In summary, this study characterizes the PEG-responsive expression profiles of *StnsLTP* genes based on qPCR results. These findings help understand the evolution and functional divergence of the potato *StnsLTP* family, and provide candidate gene resources for further investigation of potato drought response pathways.

### 4.5. Limitations and Future Perspectives

This study systematically identified the potato *nsLTP* gene family at the whole-genome level, and conducted a preliminary analysis of its evolutionary characteristics, gene structure, cis-acting elements, and expression patterns under drought stress, laying a foundation for exploring the functions of potato *StnsLTP* genes in drought stress response. However, this study still has certain limitations: the research is mainly based on bioinformatics analysis and transcriptional expression verification, and no transgenic functional validation has been performed for the screened key genes, so the specific molecular mechanisms by which these genes regulate drought stress remain unclear. Meanwhile, the expression analysis was only conducted under PEG-simulated drought treatment, without validation experiments in the field under real drought conditions. In view of the above shortcomings, future work will select the key *StnsLTP* genes with significant drought response identified in this study, perform functional verification via transgenic overexpression, CRISPR/Cas9 (Clustered regularly interspaced short palindromic repeats/CRISPR-associated protein 9) gene editing and other methods to clarify their molecular mechanisms regulating plant drought tolerance, and further evaluate the application value of the target genes in actual production combined with field drought identification experiments, so as to provide reliable gene resources and theoretical basis for molecular breeding of drought-resistant potato.

## 5. Conclusions

This study performed genome-wide identification and analysis of the *StnsLTP* gene family in potato, and clarified the chromosomal distribution, gene structure, and evolutionary characteristics of 123 *StnsLTP* genes. Tandem duplication was identified as the main driver of the expansion of this gene family, which is closely related to the *nsLTP* genes of tomato. Promoter analysis revealed that the promoter regions contain multiple elements associated with stress responses. Expression analysis under PEG-simulated drought showed that *StnsLTP.115*, *StnsLTP.117*, and *StnsLTP.118* exhibited cultivar-specific and tissue-specific expression patterns, suggesting these three genes are potential candidates associated with potato drought stress responses. This study provides basic data and gene resources for further functional exploration of *StnsLTP* genes, potato stress resistance breeding, and dissection of drought-resistant molecular mechanisms, and identifies candidate targets for future genetic improvement of stress tolerance in potato.

## Figures and Tables

**Table 1:** RT-qPCR primers and internal reference gene sequence table.

Primer Name	Primer Sequence (5′-3′)	Fragment Length (bp)
StnsLTP.115-F	GGCGAGGTTACATCTGGCTT	126
StnsLTP.115-R	TGTTTTCCGGTCTTCTGGGG	
StnsLTP.117-F	GATGAACCGTGGTCCTCTCG	113
StnsLTP.117-R	TAGCGGCTGATTTCAGGCAA	
StnsLTP.118-F	CTACCGCGGATCGAAAGACA	138
StnsLTP.118-R	CAGTGGAGGGGCTGATCTTG	
*Actin*	ATTACCGCTCTTGCTCCCAG	120
*Actin*	CCACATCTGTTGGAAGGTGC	

Note: *StnsLTP.115*, *StnsLTP.117*, *StnsLTP.118* are the target genes; *Actin* was used as the internal reference gene.

**Table 2:** Number of collinear *nsLTP* gene pairs between potato and other plant species.

Species Pair	Number of Collinear *nsLTP* Gene Pairs
*Solanum tuberosum* vs. *Solanum lycopersicum*	104
*Solanum tuberosum* vs. *Nicotiana tabacum*	73
*Solanum tuberosum* vs. *Arabidopsis thaliana*	45
*Solanum tuberosum* vs. *Oryza sativa*	23
*Solanum tuberosum* vs. *Zea mays*	15

Note: Collinearity analysis was performed using MCScanX software with default parameters.

## Data Availability

The raw data supporting the conclusions of this article will be made available by the authors, without undue reservation.

## Supplementary Materials

The following supporting information can be downloaded at: https://www.mdpi.com/article/10.3390/biology15211947/s1, Table S1. Physicochemical Properties and Subcellular Localization of StnsLTP Proteins in Potato. Table S2. Secondary structure prediction of StnsLTP protein. Table S3. Chromosomal localization of *StnsLTP* genes in potato. Table S4. Categories and counts of cis-acting elements. Table S5. Selective pressure analysis of duplicated gene pairs in the potato StnsLTP gene family and inter-species orthologous gene pairs. Table S6. Gene Ontology (GO) enrichment analysis of potato *StnsLTP* family genes. Table S7. Correspondence between *nsLTP* genes identified in the previous study (DM v4.03 genome) and *nsLTP* genes identified in this study (DM v6.1 genome). Figure S1. (A–G) Transcriptional regulatory network of the potato *StnsLTP* gene family.
