# Supplementary material for: Genome-Wide Characterization and Expression Analysis of the nsLTP Gene Family in Potato (Solanum tuberosum) in Response to Drought Stress
Source: Biology (Basel). 2026 Oct 28;15(21):1947. doi: 10.3390/biology15211947 (PMC13761951; doi:10.3390/biology15211947)
Supplement: Supplementary file 1 [file biology-15-01947-s001.zip › Supplementary Table/Supplementary Table S1.pdf]

Supplementary Table S1 Physicochemical Properties and Subcellular Localization of StnsLTP Proteins in Potato

Gene ID	Gene Name	GRAVY	Protein Length (aa)	Molecular Weight (kDa)	Theoretical pI	Instability Index	Aliphatic Index	Transmembrane Domain	Subcellular Localization
StnsLTP.001	Soltu.DM.01G001550.1	0.188	190	20.25559	7.51	52.83	95.53	2	Cell membrane
StnsLTP.002	Soltu.DM.01G021910.1	0.390	103	10.77177	7.48	41.22	100.29	1	Cell membrane
StnsLTP.003	Soltu.DM.01G027870.1	0.022	325	34.68634	5.05	39.60	66.95	0	cytoplasm
StnsLTP.004	Soltu.DM.01G028630.1	0.280	193	20.30568	7.99	68.27	98.08	0	Cell membrane
StnsLTP.005	Soltu.DM.01G028720.1	0.417	121	12.39160	8.62	56.78	100.00	1	Cell membrane；Cell wall
StnsLTP.006	Soltu.DM.01G028730.1	0.296	120	13.05230	9.20	57.27	87.83	1	Cell wall
StnsLTP.007	Soltu.DM.01G031010.1	0.248	118	12.14108	8.14	31.18	97.63	1	Cell membrane; Cell wall
StnsLTP.008	Soltu.DM.01G031020.1	0.322	113	11.53739	8.47	28.13	98.41	1	Cell wall
StnsLTP.009	Soltu.DM.01G031610.1	0.665	122	12.76441	8.98	26.20	107.05	0	Cell membrane
StnsLTP.010	Soltu.DM.01G034890.1	-0.087	112	12.36966	9.53	28.58	85.36	1	Cell wall
StnsLTP.011	Soltu.DM.01G042060.1	0.218	137	14.80961	8.11	51.20	80.44	1	Cell membrane
StnsLTP.012	Soltu.DM.01G049040.1	0.330	104	10.94996	7.47	36.92	86.44	1	Cell membrane
StnsLTP.013	Soltu.DM.02G026200.1	0.303	96	10.02483	8.58	38.39	75.10	0	Cell wall
StnsLTP.014	Soltu.DM.02G031780.1	0.073	109	11.23001	8.14	37.87	81.47	0	Cell wall
StnsLTP.015	Soltu.DM.03G000250.1	0.087	168	17.15047	8.71	61.93	77.86	2	Cell membrane
StnsLTP.016	Soltu.DM.03G000280.1	0.289	209	21.13437	8.44	58.80	72.58	2	Cell membrane
StnsLTP.017	Soltu.DM.03G004560.1	0.660	97	10.37556	7.47	37.67	110.52	1	Cell membrane
StnsLTP.018	Soltu.DM.03G004620.1	0.688	97	10.34853	7.47	37.67	110.52	1	Cell membrane
StnsLTP.019	Soltu.DM.03G004630.1	0.250	92	9.94775	8.88	55.56	87.93	1	Cell membrane
StnsLTP.020	Soltu.DM.03G004670.1	0.481	96	10.64966	7.48	39.46	82.19	1	Cell membrane
StnsLTP.021	Soltu.DM.03G004690.1	0.123	104	11.43282	9.41	20.95	86.15	0	Cell membrane;

									Cell wall
StnsLTP.022	Soltu.DM.03G012340.1	0.172	112	11.67866	8.08	33.04	88.93	0	Cell membrane
StnsLTP.023	Soltu.DM.03G015150.1	0.739	125	12.63091	7.43	21.49	120.08	1	Cell membrane
StnsLTP.024	Soltu.DM.03G015160.1	0.274	205	18.20420	8.38	52.79	71.37	1	Nucleus
StnsLTP.025	Soltu.DM.03G015170.3	0.268	207	18.31830	8.38	53.56	70.68	1	Nucleus
StnsLTP.026	Soltu.DM.03G015190.1	0.268	207	18.31830	8.38	53.56	70.68	1	Nucleus
StnsLTP.027	Soltu.DM.03G015220.1	0.712	120	12.28342	5.90	37.06	121.83	1	Cell membrane
StnsLTP.028	Soltu.DM.03G015230.1	0.712	120	12.28342	5.90	37.06	121.83	1	Cell membrane
StnsLTP.029	Soltu.DM.03G015240.1	0.712	120	12.28342	5.90	37.06	121.83	1	Cell membrane
StnsLTP.030	Soltu.DM.03G017060.1	0.530	112	11.66144	4.16	25.57	83.57	0	Cell wall
StnsLTP.031	Soltu.DM.03G032810.1	-0.237	84	9.24596	8.78	64.34	61.67	0	Cell membrane
StnsLTP.032	Soltu.DM.03G032930.1	0.389	161	16.65856	8.34	37.65	81.24	1	Cell membrane
StnsLTP.033	Soltu.DM.03G033630.1	0.365	93	9.87983	9.44	37.66	104.84	1	Cell membrane; Cell wall
StnsLTP.034	Soltu.DM.03G034110.1	0.363	185	19.54347	5.05	45.63	92.22	0	Cell membrane
StnsLTP.035	Soltu.DM.03G036710.1	0.158	106	11.34632	8.15	48.52	93.02	0	Cell membrane
StnsLTP.036	Soltu.DM.05G011870.1	0.101	197	20.42835	5.63	52.78	91.62	2	Cell membrane
StnsLTP.037	Soltu.DM.05G018410.1	-0.171	160	17.68162	9.49	59.99	100.56	0	Nucleus
StnsLTP.038	Soltu.DM.06G003700.1	0.397	117	12.67834	9.48	50.75	105.04	1	Cell wall
StnsLTP.039	Soltu.DM.06G003710.1	0.209	118	12.89240	9.11	30.46	94.92	1	Cell wall
StnsLTP.040	Soltu.DM.06G003720.1	0.288	117	12.74918	8.60	60.17	98.29	1	Cell wall
StnsLTP.041	Soltu.DM.06G003730.1	0.319	119	12.67586	8.08	38.04	98.24	0	Cell wall
StnsLTP.042	Soltu.DM.06G013400.1	0.109	128	13.60805	8.93	33.59	90.00	1	Cell membrane
StnsLTP.043	Soltu.DM.06G015050.1	0.162	208	21.94770	9.42	67.62	88.12	1	Cell membrane
StnsLTP.044	Soltu.DM.06G015070.1	0.094	153	15.68799	6.11	74.25	73.40	1	Cell membrane
StnsLTP.045	Soltu.DM.06G015080.1	0.201	147	15.58574	4.43	56.08	85.65	2	Cell membrane

StnsLTP.046	Soltu.DM.06G016150.1	0.070	101	10.76455	4.23	37.30	77.43	0	Cell wall
StnsLTP.047	Soltu.DM.06G016170.1	0.264	118	12.86099	4.81	47.29	93.47	1	Cell wall
StnsLTP.048	Soltu.DM.06G017790.1	0.509	134	14.13283	9.03	33.64	112.91	1	Cell membrane
StnsLTP.049	Soltu.DM.06G019440.1	0.771	129	13.26898	8.35	29.05	117.91	0	Cell membrane
StnsLTP.050	Soltu.DM.06G025000.1	0.086	94	10.20338	9.77	63.82	98.51	1	Cell membrane
StnsLTP.051	Soltu.DM.06G025060.1	0.158	180	18.73782	5.40	48.58	93.28	0	Cell membrane
StnsLTP.052	Soltu.DM.06G029040.1	0.344	194	20.13740	5.22	64.89	93.04	1	Cell membrane
StnsLTP.053	Soltu.DM.06G034620.1	0.341	184	18.17363	8.16	57.10	85.87	1	Cell membrane
StnsLTP.054	Soltu.DM.06G034630.1	0.317	151	15.51296	8.40	38.84	83.38	0	Cell membrane
StnsLTP.055	Soltu.DM.07G013810.1	-0.199	390	41.22451	9.67	65.89	83.10	0	Cell wall
StnsLTP.056	Soltu.DM.07G020060.1	-0.055	125	14.42562	6.49	46.17	90.48	1	Cell wall
StnsLTP.057	Soltu.DM.07G025650.1	-0.412	141	16.18058	5.91	48.52	75.39	0	Endoplasmic Reticulum;Vacuole
StnsLTP.058	Soltu.DM.07G025730.1	-0.637	224	26.19011	6.55	57.19	74.91	0	Nucleus
StnsLTP.059	Soltu.DM.07G026820.1	0.404	102	10.89335	9.28	20.58	91.86	1	Cell membrane
StnsLTP.060	Soltu.DM.08G000480.1	0.359	150	15.35079	4.56	56.09	91.20	0	Cell membrane
StnsLTP.061	Soltu.DM.08G002860.1	0.543	138	14.61366	9.11	31.24	105.87	1	Cell membrane
StnsLTP.062	Soltu.DM.08G016080.1	-0.506	345	37.52862	8.89	40.79	73.54	0	Cell membrane; Nucleus
StnsLTP.063	Soltu.DM.08G016090.1	0.623	122	12.74340	9.46	23.89	107.05	1	Cell wall
StnsLTP.064	Soltu.DM.08G016100.1	0.294	125	13.16431	7.45	34.18	99.12	0	Cell wall
StnsLTP.065	Soltu.DM.08G016140.1	0.192	121	13.04829	9.34	26.78	92.73	0	Cell wall
StnsLTP.066	Soltu.DM.08G016150.1	0.516	116	11.81381	8.42	40.83	104.40	0	Cell wall
StnsLTP.067	Soltu.DM.08G016160.1	-0.101	114	12.28828	9.30	46.28	88.25	0	Cell wall
StnsLTP.068	Soltu.DM.08G016180.1	0.504	128	13.30070	8.94	52.48	103.59	1	Cell wall
StnsLTP.069	Soltu.DM.08G018950.1	0.674	133	13.86272	8.43	43.75	108.57	1	Cell membrane

StnsLTP.070	Soltu.DM.08G023700.1	0.080	114	12.81339	9.32	55.23	92.28	0	Chloroplast; Nucleus
StnsLTP.071	Soltu.DM.08G023710.1	0.145	123	13.83166	9.47	31.72	98.13	1	Chloroplast; Nucleus
StnsLTP.072	Soltu.DM.08G025180.1	0.182	153	16.06195	9.02	40.66	94.38	1	Cell membrane
StnsLTP.073	Soltu.DM.08G025200.1	0.158	284	28.72924	9.01	74.10	88.20	1	Cell membrane; Nucleus
StnsLTP.074	Soltu.DM.08G025210.1	0.367	138	14.26332	9.55	39.34	107.39	1	Cell membrane
StnsLTP.075	Soltu.DM.08G025220.1	0.583	132	13.79465	8.87	33.73	113.03	1	Cell membrane
StnsLTP.076	Soltu.DM.08G025230.1	0.267	141	14.87861	8.76	51.65	101.70	1	Cell membrane
StnsLTP.077	Soltu.DM.08G025240.1	0.563	131	13.78680	9.12	34.57	116.11	1	Cell membrane
StnsLTP.078	Soltu.DM.08G025250.1	0.371	141	14.94273	8.36	39.33	104.47	1	Cell membrane
StnsLTP.079	Soltu.DM.08G025480.1	0.229	143	15.01374	8.76	45.94	99.58	0	Cell membrane
StnsLTP.080	Soltu.DM.08G025490.1	0.319	139	14.55818	8.39	50.59	104.60	0	Cell membrane
StnsLTP.081	Soltu.DM.08G025500.1	0.324	139	14.73440	8.08	51.64	107.41	1	Cell membrane
StnsLTP.082	Soltu.DM.08G025530.1	0.187	119	12.35930	5.17	50.77	90.25	0	Cell membrane
StnsLTP.083	Soltu.DM.08G025540.1	0.374	151	15.64223	8.12	47.82	93.77	0	Cell membrane
StnsLTP.084	Soltu.DM.08G027140.1	-0.492	144	16.57001	8.11	63.55	76.53	1	Vacuole
StnsLTP.085	Soltu.DM.09G005000.1	0.902	101	10.74683	5.33	40.49	122.57	0	Cell wall
StnsLTP.086	Soltu.DM.09G010750.1	0.425	110	11.10606	9.00	36.06	87.91	0	Cell wall
StnsLTP.087	Soltu.DM.09G019990.1	0.229	125	12.93687	8.50	47.81	91.28	0	Cell membrane
StnsLTP.088	Soltu.DM.09G020000.2	0.242	173	17.37261	7.49	52.54	76.71	0	Cell membrane
StnsLTP.089	Soltu.DM.09G020010.1	0.320	185	18.33476	4.66	52.82	86.00	2	Cell membrane
StnsLTP.090	Soltu.DM.09G020020.1	0.101	253	26.72092	7.61	54.44	87.47	1	Cell membrane
StnsLTP.091	Soltu.DM.09G020300.1	0.230	129	14.20194	8.54	41.91	108.76	1	Cell wall
StnsLTP.092	Soltu.DM.09G024650.1	0.180	164	16.80296	4.39	41.61	77.99	0	Cell membrane

StnsLTP.093	Soltu.DM.09G024660.1	0.585	174	17.22912	8.41	51.03	93.10	1	Cell membrane
StnsLTP.094	Soltu.DM.09G024670.1	0.191	221	22.87975	5.78	55.66	77.74	1	Cell membrane
StnsLTP.095	Soltu.DM.09G024680.1	-0.073	235	24.22462	8.38	59.94	69.70	1	Cell membrane; Nucleus
StnsLTP.096	Soltu.DM.09G024690.1	0.076	208	22.11719	4.08	61.06	89.57	0	Cell membrane
StnsLTP.097	Soltu.DM.10G006020.1	0.184	179	19.36177	6.67	36.68	94.30	0	Cell wall
StnsLTP.098	Soltu.DM.10G006030.1	0.337	112	11.90118	8.44	35.95	96.79	0	Cell wall
StnsLTP.099	Soltu.DM.10G006050.1	0.180	112	11.99228	8.99	28.36	93.21	0	Cell wall
StnsLTP.100	Soltu.DM.10G006060.1	0.171	115	12.54772	6.69	45.28	96.70	0	Cell wall
StnsLTP.101	Soltu.DM.10G006090.1	0.316	115	12.38276	8.65	34.75	104.35	0	Cell wall
StnsLTP.102	Soltu.DM.10G007820.1	0.045	118	13.16251	7.52	40.25	100.76	0	Cell wall
StnsLTP.103	Soltu.DM.10G018660.1	0.633	120	12.08249	8.72	32.97	103.25	1	Cell wall
StnsLTP.104	Soltu.DM.10G018670.1	0.475	113	11.58369	8.89	49.79	90.53	0	Cell wall
StnsLTP.105	Soltu.DM.10G018680.1	0.430	114	11.44363	8.87	34.61	89.91	0	Cell wall
StnsLTP.106	Soltu.DM.10G018690.1	0.514	114	11.37860	8.87	39.48	97.54	0	Cell wall
StnsLTP.107	Soltu.DM.10G018700.1	0.592	118	12.00643	9.10	35.73	99.24	0	Cell membrane; Cell wall
StnsLTP.108	Soltu.DM.10G018710.1	0.616	116	11.74612	9.21	36.18	100.95	0	Cell wall
StnsLTP.109	Soltu.DM.10G018740.1	0.588	118	12.02046	9.10	35.73	100.08	0	Cell wall
StnsLTP.110	Soltu.DM.10G018750.1	0.635	116	11.64196	8.98	46.81	105.17	0	Cell wall
StnsLTP.111	Soltu.DM.10G018760.1	0.529	116	11.46064	8.87	43.49	98.45	1	Cell wall
StnsLTP.112	Soltu.DM.10G018770.1	0.537	112	11.06021	9.00	44.69	97.59	0	Cell wall
StnsLTP.113	Soltu.DM.10G018780.1	0.533	114	11.33454	8.87	40.23	97.54	0	Cell wall
StnsLTP.114	Soltu.DM.10G018790.1	0.412	114	11.40549	8.34	40.48	88.16	0	Cell membrane; Cell wall
StnsLTP.115	Soltu.DM.10G018810.1	0.459	114	11.36043	8.34	38.32	91.58	0	Cell membrane;

									Cell wall
StnsLTP.116	Soltu.DM.10G018820.1	0.459	114	11.36043	8.34	38.32	91.58	0	Cell membrane; Cell wall
StnsLTP.117	Soltu.DM.10G018830.1	0.414	114	11.64967	8.36	42.95	88.16	0	Cell wall
StnsLTP.118	Soltu.DM.10G018840.1	0.510	114	11.47164	8.73	39.04	95.88	0	Cell membrane; Cell wall
StnsLTP.119	Soltu.DM.10G018860.1	0.398	121	12.78621	9.10	49.57	92.64	0	Cell wall
StnsLTP.120	Soltu.DM.10G018870.1	0.157	101	10.71380	9.30	19.55	93.56	0	Cell wall
StnsLTP.121	Soltu.DM.11G025020.1	0.370	206	21.28884	4.49	57.47	89.13	0	Cell membrane
StnsLTP.122	Soltu.DM.12G026740.1	0.529	241	24.92167	9.20	52.28	121.16	0	Cell membrane; Nucleus
StnsLTP.123	Soltu.DM.S000070.1	0.463	138	14.63764	9.76	31.96	100.58	1	Cell membrane

Note: Transmembrane helices were predicted using TMHMM 2.0 online tools; values indicate the number of transmembrane domains (0 = no helix, 1 = one helix, 2 = two helices).
Physicochemical properties (including GRAVY, molecular weight, theoretical pI, instability index, and aliphatic index) were calculated using the ProtParam tool (ExPASy).
Subcellular localization was predicted using the Cell-PLoc 2.0.
