# Supplementary material for: Genome-Wide Characterization and Expression Analysis of the nsLTP Gene Family in Potato (Solanum tuberosum) in Response to Drought Stress
Source: Biology (Basel). 2026 Oct 28;15(21):1947. doi: 10.3390/biology15211947 (PMC13761951; doi:10.3390/biology15211947)
Supplement: Supplementary file 1 [file biology-15-01947-s001.zip › Supplementary Table/Supplementary Table S2.pdf]

Supplementary Table S2 Secondary structure prediction of StnsLTP protein

Gene ID	α-helix （%）	Extended strand （%）	β-turn （%）	Random coil (%)	Gene ID	α-helix (%)	Extended strand (%)	β-turn (%)	Random coil (%)
StnsLTP.001	34.21	8.95	0	56.84	*StnsLTP.063*	50.00	9.02	1.64	39.34
StnsLTP.002	43.69	3.88	0	52.43	*StnsLTP.064*	48.80	7.20	0.80	43.20
StnsLTP.003	9.54	24.31	4.92	61.23	*StnsLTP.065*	50.41	7.44	0	42.15
StnsLTP.004	35.23	6.74	1.55	56.48	*StnsLTP.066*	44.83	9.48	1.72	43.97
StnsLTP.005	49.59	7.44	0.83	42.15	*StnsLTP.067*	50.00	7.89	0.88	41.23
StnsLTP.006	45.00	8.33	0.83	45.83	*StnsLTP.068*	52.34	7.03	0	40.62
StnsLTP.007	48.31	6.78	0.85	44.07	*StnsLTP.069*	39.10	10.53	1.50	48.87
StnsLTP.008	48.67	6.19	0.88	44.25	*StnsLTP.070*	50.00	7.02	1.75	41.23
StnsLTP.009	45.08	12.3	0	42.62	*StnsLTP.071*	56.10	5.69	1.63	36.59
StnsLTP.010	41.07	8.04	0.89	50.00	*StnsLTP.072*	33.99	8.50	1.31	56.21
StnsLTP.011	40.15	3.65	0	56.20	*StnsLTP.073*	12.68	3.87	0.70	82.75
StnsLTP.012	50.00	0.06	0	44.23	*StnsLTP.074*	40.58	11.59	1.45	46.38
StnsLTP.013	43.75	3.12	0	53.12	*StnsLTP.075*	31.82	12.88	0	55.30
StnsLTP.014	45.87	6.42	0.92	46.79	*StnsLTP.076*	38.30	9.93	1.42	50.35
StnsLTP.015	37.50	6.55	0	55.95	*StnsLTP.077*	41.98	12.21	1.53	44.27
StnsLTP.016	23.92	4.31	0	71.29	*StnsLTP.078*	41.84	12.77	1.42	43.97
StnsLTP.017	46.39	4.12	2.06	47.42	*StnsLTP.079*	40.56	9.09	1.40	48.95
StnsLTP.018	45.36	4.12	2.06	48.45	*StnsLTP.080*	41.01	10.79	1.44	46.76
StnsLTP.019	44.57	4.35	0	51.09	*StnsLTP.081*	40.29	10.07	1.44	48.20
StnsLTP.020	51.04	4.17	6.25	38.54	*StnsLTP.082*	40.34	3.36	0	56.30
StnsLTP.021	37.50	3.85	1.92	56.73	*StnsLTP.083*	39.07	3.97	0	56.95
StnsLTP.022	36.61	7.14	0	56.25	*StnsLTP.084*	62.50	6.25	0	31.25
StnsLTP.023	43.20	15.20	1.6	40.00	*StnsLTP.085*	40.59	13.86	0.99	44.55
StnsLTP.024	27.80	10.73	0.98	60.49	*StnsLTP.086*	48.18	8.18	0.91	42.73
StnsLTP.025	24.15	12.08	0.97	62.80	*StnsLTP.087*	31.20	8.80	0.80	59.20
StnsLTP.026	24.15	12.08	0.97	62.80	*StnsLTP.088*	35.84	7.51	0.58	56.07
StnsLTP.027	44.17	12.50	1.67	41.67	*StnsLTP.089*	29.19	9.19	0.54	61.08
StnsLTP.028	44.17	12.50	1.67	41.67	*StnsLTP.090*	19.76	7.11	0	73.12
StnsLTP.029	44.17	12.50	1.67	41.67	*StnsLTP.091*	47.29	7.75	0.78	44.19
StnsLTP.030	42.86	9.82	0	47.32	*StnsLTP.092*	34.15	6.71	1.83	57.32
StnsLTP.031	39.29	7.14	0	53.57	*StnsLTP.093*	36.78	8.62	2.30	52.30
StnsLTP.032	49.69	3.11	0	47.20	*StnsLTP.094*	16.29	5.88	0	77.83
StnsLTP.033	47.31	3.23	1.08	48.39	*StnsLTP.095*	24.68	7.23	0.85	67.23
StnsLTP.034	36.22	7.03	0	56.76	*StnsLTP.096*	23.56	7.69	0	68.75
StnsLTP.035	49.06	3.77	0	47.17	*StnsLTP.097*	48.60	5.59	2.23	43.58
StnsLTP.036	32.99	7.61	0.51	58.88	*StnsLTP.098*	42.86	8.04	0.89	48.21
StnsLTP.037	36.88	11.88	0	51.25	*StnsLTP.099*	44.64	8.04	0.89	46.43
StnsLTP.038	44.44	8.55	3.42	43.59	*StnsLTP.100*	43.48	6.96	0	49.57
StnsLTP.039	44.92	8.47	1.69	44.92	*StnsLTP.101*	43.48	7.83	0	48.70
StnsLTP.040	48.72	9.40	1.71	40.17	*StnsLTP.102*	49.15	6.78	0	44.07
StnsLTP.041	45.38	9.24	1.68	43.70	*StnsLTP.103*	47.50	10.83	1.67	40.00
StnsLTP.042	51.56	3.91	0	44.53	*StnsLTP.104*	47.79	9.73	0.88	41.59
StnsLTP.043	27.88	6.73	0.48	64.90	*StnsLTP.105*	47.37	7.89	0	44.74
StnsLTP.044	33.33	6.54	1.96	58.17	*StnsLTP.106*	49.12	9.65	1.75	39.47
StnsLTP.045	31.97	6.80	0.68	60.54	*StnsLTP.107*	47.46	11.86	0	40.68
StnsLTP.046	39.60	12.87	0.99	46.53	*StnsLTP.108*	43.97	13.79	0.86	41.38
StnsLTP.047	45.76	5.93	0.85	47.46	*StnsLTP.109*	45.76	11.86	0.85	41.53

StnsLTP.048	41.79	11.94	1.49	44.78	*StnsLTP.110*	46.55	12.93	1.72	38.79
StnsLTP.049	40.31	12.40	1.55	45.74	*StnsLTP.111*	49.14	8.62	0.86	41.38
StnsLTP.050	43.62	6.38	6.38	43.62	*StnsLTP.112*	49.11	8.93	1.79	40.18
StnsLTP.051	26.67	0.07	2.22	63.89	*StnsLTP.113*	46.49	9.65	0.88	42.98
StnsLTP.052	36.60	5.15	1.55	56.70	*StnsLTP.114*	46.49	10.53	1.75	41.23
StnsLTP.053	32.61	6.52	2.17	58.70	*StnsLTP.115*	46.49	9.65	0.88	42.98
StnsLTP.054	27.81	6.62	2.65	62.91	*StnsLTP.116*	46.49	9.65	0.88	42.98
StnsLTP.055	11.03	1.79	1.03	86.15	*StnsLTP.117*	47.37	8.77	0.88	42.98
StnsLTP.056	43.20	8.00	0	48.80	*StnsLTP.118*	49.12	9.65	0.88	40.35
StnsLTP.057	59.57	6.38	0	34.04	*StnsLTP.119*	43.80	11.57	0.83	43.80
StnsLTP.058	64.73	6.70	0.89	27.68	*StnsLTP.120*	38.61	13.86	0.99	46.53
StnsLTP.059	45.10	4.90	0	50.00	*StnsLTP.121*	29.61	4.37	1.94	64.08
StnsLTP.060	42.00	4.67	0	53.33	*StnsLTP.122*	14.94	4.56	0	80.50
StnsLTP.061	39.13	11.59	1.45	47.83	*StnsLTP.123*	39.13	5.80	0	55.07
StnsLTP.062	32.17	3.77	3.19	60.87					

Note: Secondary structure components (α-helix, extended strand, β-turn, and random coil) were predicted using the SOPMA online tool with default parameters.
