# Supplementary material for: Genome-Wide Characterization and Expression Analysis of the nsLTP Gene Family in Potato (Solanum tuberosum) in Response to Drought Stress
Source: Biology (Basel). 2026 Oct 28;15(21):1947. doi: 10.3390/biology15211947 (PMC13761951; doi:10.3390/biology15211947)
Supplement: Supplementary file 1 [file biology-15-01947-s001.zip › Supplementary Table/Supplementary Table S3.pdf]

Supplementary Table S3 Chromosomal localization of *StnsLTP* genes in potato

Gene ID	Gene Name	Chromosome	Start Position (bp)	End Position (bp)
StnsLTP.001	Soltu.DM.01G001550.1	chr01	1605488	1607218
StnsLTP.002	Soltu.DM.01G021910.1	chr01	59670317	59670890
StnsLTP.003	Soltu.DM.01G027870.1	chr01	67674006	67678249
StnsLTP.004	Soltu.DM.01G028630.1	chr01	68398984	68400630
StnsLTP.005	Soltu.DM.01G028720.1	chr01	68478965	68480451
StnsLTP.006	Soltu.DM.01G028730.1	chr01	68480972	68481905
StnsLTP.007	Soltu.DM.01G031010.1	chr01	70764496	70764852
StnsLTP.008	Soltu.DM.01G031020.1	chr01	70772337	70772678
StnsLTP.009	Soltu.DM.01G031610.1	chr01	71464002	71464793
StnsLTP.010	Soltu.DM.01G034890.1	chr01	74432708	74433236
StnsLTP.011	Soltu.DM.01G042060.1	chr01	80436248	80436912
StnsLTP.012	Soltu.DM.01G049040.1	chr01	85958673	85959285
StnsLTP.013	Soltu.DM.02G026200.1	chr02	39230438	39230728
StnsLTP.014	Soltu.DM.02G031780.1	chr02	43784004	43784333
StnsLTP.015	Soltu.DM.03G000250.1	chr03	356308	357657
StnsLTP.016	Soltu.DM.03G000280.1	chr03	361828	364016
StnsLTP.017	Soltu.DM.03G004560.1	chr03	4831198	4831491
StnsLTP.018	Soltu.DM.03G004620.1	chr03	4974987	4975280
StnsLTP.019	Soltu.DM.03G004630.1	chr03	5031339	5031900
StnsLTP.020	Soltu.DM.03G004670.1	chr03	5069394	5069982
StnsLTP.021	Soltu.DM.03G004690.1	chr03	5090124	5090742
StnsLTP.022	Soltu.DM.03G012340.1	chr03	33449649	33450365
StnsLTP.023	Soltu.DM.03G015150.1	chr03	38578016	38578927
StnsLTP.024	Soltu.DM.03G015160.1	chr03	38582884	38583795
StnsLTP.025	Soltu.DM.03G015170.3	chr03	38623533	38624450
StnsLTP.026	Soltu.DM.03G015190.1	chr03	38648734	38649357
StnsLTP.027	Soltu.DM.03G015220.1	chr03	38715277	38715639
StnsLTP.028	Soltu.DM.03G015230.1	chr03	38730803	38731165
StnsLTP.029	Soltu.DM.03G015240.1	chr03	38748387	38748749
StnsLTP.030	Soltu.DM.03G017060.1	chr03	41453331	41453669
StnsLTP.031	Soltu.DM.03G032810.1	chr03	56612734	56614137
StnsLTP.032	Soltu.DM.03G032930.1	chr03	56682958	56684470
StnsLTP.033	Soltu.DM.03G033630.1	chr03	57230835	57231116
StnsLTP.034	Soltu.DM.03G034110.1	chr03	57563855	57564734
StnsLTP.035	Soltu.DM.03G036710.1	chr03	59630878	59631511
StnsLTP.036	Soltu.DM.05G011870.1	chr05	14194144	14198184
StnsLTP.037	Soltu.DM.05G018410.1	chr05	45373221	45374093
StnsLTP.038	Soltu.DM.06G003700.1	chr06	4437985	4438546
StnsLTP.039	Soltu.DM.06G003710.1	chr06	4456713	4457419
StnsLTP.040	Soltu.DM.06G003720.1	chr06	4466326	4466974
StnsLTP.041	Soltu.DM.06G003730.1	chr06	4474608	4474967
StnsLTP.042	Soltu.DM.06G013400.1	chr06	38344505	38345225

StnsLTP.043	Soltu.DM.06G015050.1	chr06	40713261	40714361
StnsLTP.044	Soltu.DM.06G015070.1	chr06	40739823	40740847
StnsLTP.045	Soltu.DM.06G015080.1	chr06	40741349	40742339
StnsLTP.046	Soltu.DM.06G016150.1	chr06	41780069	41780374
StnsLTP.047	Soltu.DM.06G016170.1	chr06	41787629	41787985
StnsLTP.048	Soltu.DM.06G017790.1	chr06	44042624	44043412
StnsLTP.049	Soltu.DM.06G019440.1	chr06	46042585	46043429
StnsLTP.050	Soltu.DM.06G025000.1	chr06	50969601	50969885
StnsLTP.051	Soltu.DM.06G025060.1	chr06	51001348	51001993
StnsLTP.052	Soltu.DM.06G029040.1	chr06	54243493	54246241
StnsLTP.053	Soltu.DM.06G034620.1	chr06	58605705	58607033
StnsLTP.054	Soltu.DM.06G034630.1	chr06	58608457	58609501
StnsLTP.055	Soltu.DM.07G013810.1	chr07	42923548	42925566
StnsLTP.056	Soltu.DM.07G020060.1	chr07	50575137	50575611
StnsLTP.057	Soltu.DM.07G025650.1	chr07	55315533	55316520
StnsLTP.058	Soltu.DM.07G025730.1	chr07	55411915	55414475
StnsLTP.059	Soltu.DM.07G026820.1	chr07	56151744	56152338
StnsLTP.060	Soltu.DM.08G000480.1	chr08	1004487	1007060
StnsLTP.061	Soltu.DM.08G002860.1	chr08	3437200	3437937
StnsLTP.062	Soltu.DM.08G016080.1	chr08	44067376	44071937
StnsLTP.063	Soltu.DM.08G016090.1	chr08	44079558	44080312
StnsLTP.064	Soltu.DM.08G016100.1	chr08	44082038	44082526
StnsLTP.065	Soltu.DM.08G016140.1	chr08	44114032	44114397
StnsLTP.066	Soltu.DM.08G016150.1	chr08	44116964	44117537
StnsLTP.067	Soltu.DM.08G016160.1	chr08	44127770	44130078
StnsLTP.068	Soltu.DM.08G016180.1	chr08	44143321	44143792
StnsLTP.069	Soltu.DM.08G018950.1	chr08	47745571	47746445
StnsLTP.070	Soltu.DM.08G023700.1	chr08	53345874	53346958
StnsLTP.071	Soltu.DM.08G023710.1	chr08	53349259	53350654
StnsLTP.072	Soltu.DM.08G025180.1	chr08	55005336	55006126
StnsLTP.073	Soltu.DM.08G025200.1	chr08	55015803	55016931
StnsLTP.074	Soltu.DM.08G025210.1	chr08	55019516	55020275
StnsLTP.075	Soltu.DM.08G025220.1	chr08	55025011	55025782
StnsLTP.076	Soltu.DM.08G025230.1	chr08	55030934	55031629
StnsLTP.077	Soltu.DM.08G025240.1	chr08	55032889	55033685
StnsLTP.078	Soltu.DM.08G025250.1	chr08	55036543	55037456
StnsLTP.079	Soltu.DM.08G025480.1	chr08	55292366	55293161
StnsLTP.080	Soltu.DM.08G025490.1	chr08	55301446	55302155
StnsLTP.081	Soltu.DM.08G025500.1	chr08	55308009	55308735
StnsLTP.082	Soltu.DM.08G025530.1	chr08	55322347	55322864
StnsLTP.083	Soltu.DM.08G025540.1	chr08	55325647	55328902
StnsLTP.084	Soltu.DM.08G027140.1	chr08	56638374	56639251
StnsLTP.085	Soltu.DM.09G005000.1	chr09	4456371	4457224
StnsLTP.086	Soltu.DM.09G010750.1	chr09	16260296	16261867

StnsLTP.087	Soltu.DM.09G019990.1	chr09	54591804	54593306
StnsLTP.088	Soltu.DM.09G020000.2	chr09	54600637	54601992
StnsLTP.089	Soltu.DM.09G020010.1	chr09	54603238	54604704
StnsLTP.090	Soltu.DM.09G020020.1	chr09	54606069	54608112
StnsLTP.091	Soltu.DM.09G020300.1	chr09	54938026	54939643
StnsLTP.092	Soltu.DM.09G024650.1	chr09	60564750	60566394
StnsLTP.093	Soltu.DM.09G024660.1	chr09	60573130	60575508
StnsLTP.094	Soltu.DM.09G024670.1	chr09	60578747	60581065
StnsLTP.095	Soltu.DM.09G024680.1	chr09	60583229	60584523
StnsLTP.096	Soltu.DM.09G024690.1	chr09	60585570	60588015
StnsLTP.097	Soltu.DM.10G006020.1	chr10	6167410	6169693
StnsLTP.098	Soltu.DM.10G006030.1	chr10	6180870	6181208
StnsLTP.099	Soltu.DM.10G006050.1	chr10	6197767	6198105
StnsLTP.100	Soltu.DM.10G006060.1	chr10	6205955	6206302
StnsLTP.101	Soltu.DM.10G006090.1	chr10	6225505	6225852
StnsLTP.102	Soltu.DM.10G007820.1	chr10	10682454	10682810
StnsLTP.103	Soltu.DM.10G018660.1	chr10	49752694	49753056
StnsLTP.104	Soltu.DM.10G018670.1	chr10	49755243	49755584
StnsLTP.105	Soltu.DM.10G018680.1	chr10	49758302	49759170
StnsLTP.106	Soltu.DM.10G018690.1	chr10	49763420	49764277
StnsLTP.107	Soltu.DM.10G018700.1	chr10	49767119	49767475
StnsLTP.108	Soltu.DM.10G018710.1	chr10	49783703	49784053
StnsLTP.109	Soltu.DM.10G018740.1	chr10	49858256	49858612
StnsLTP.110	Soltu.DM.10G018750.1	chr10	49863101	49863451
StnsLTP.111	Soltu.DM.10G018760.1	chr10	49865754	49866371
StnsLTP.112	Soltu.DM.10G018770.1	chr10	49870147	49870721
StnsLTP.113	Soltu.DM.10G018780.1	chr10	49875679	49876572
StnsLTP.114	Soltu.DM.10G018790.1	chr10	49896392	49897182
StnsLTP.115	Soltu.DM.10G018810.1	chr10	49918328	49919253
StnsLTP.116	Soltu.DM.10G018820.1	chr10	49941483	49942404
StnsLTP.117	Soltu.DM.10G018830.1	chr10	49962333	49963360
StnsLTP.118	Soltu.DM.10G018840.1	chr10	49986614	49987582
StnsLTP.119	Soltu.DM.10G018860.1	chr10	50004870	50005235
StnsLTP.120	Soltu.DM.10G018870.1	chr10	50016844	50017775
StnsLTP.121	Soltu.DM.11G025020.1	chr11	45085683	45088829
StnsLTP.122	Soltu.DM.12G026740.1	chr12	56693085	56694575
StnsLTP.123	Soltu.DM.S000070.1		40921	41643

Note: Chromosomal positions of *StnsLTP* genes were retrieved from the potato reference genome using the Spud DB database.
