# Supplementary material for: Genome-Wide Characterization and Expression Analysis of the nsLTP Gene Family in Potato (Solanum tuberosum) in Response to Drought Stress
Source: Biology (Basel). 2026 Oct 28;15(21):1947. doi: 10.3390/biology15211947 (PMC13761951; doi:10.3390/biology15211947)
Supplement: Supplementary file 1 [file biology-15-01947-s001.zip › Supplementary Table/Supplementary Table S4.pdf]

Supplementary Table S4 Categories and counts of cis-acting elements

Category	Cis-element	Count
Light responsive	3-AF1 binding site, 4cl-CMA1c, AAAC-motif, ACA-motif, ACE, AE-box, AT1-motif, ATC-motif, ATCT-motif, Box4, BoxII, chs-CMA1a, chs-CMA2a, chs-CMA2b, GA-motif, Gap-box, GATA-motif, G-box, GT1-motif, GTGGC-motif, I-box, LAMP-element, L-box, LS7, MRE, Pc-CMA2a, sbp-CMA1c, Sp1, TCCC-motif, TCT-motif, circadian, A-box	32
Phytohormone responsive	ABRE, AuxRE, AuxRR-core, CGTCA-motif, GARE-motif, P-box, TATC-box, TCA-element, TGA-box, TGACG-motif, TGA-element	11
Development related	AACA_motif, CAT-box, GCN4_motif, HD-Zip1, MBSI, MSA-like, RY-element, O2-site	8
Abiotic and biotic stress	ARE, GC-motif, LTR, MBS, TC-richrepeats, WUN-motif	6
Other responsive elements	3-AF3bindingsite, AT-richelement, AT-richsequence, BoxIII, CCAAT-box, HD-Zip3	6

Note: Cis-acting elements were predicted using the PlantCARE online tool.
