# Supplementary material for: Genome-Wide Characterization and Expression Analysis of the nsLTP Gene Family in Potato (Solanum tuberosum) in Response to Drought Stress
Source: Biology (Basel). 2026 Oct 28;15(21):1947. doi: 10.3390/biology15211947 (PMC13761951; doi:10.3390/biology15211947)
Supplement: Supplementary file 1 [file biology-15-01947-s001.zip › Supplementary Table/Supplementary Table S5.pdf]

Supplementary Table S5 Selective pressure analysis of duplicated gene pairs in the potato StnsLTP gene family and inter-species orthologous gene pairs

Gene pair 1	Gene pair 2	Ka	Ks	Ka_Ks	Type
Soltu.DM.01G031010.1	Soltu.DM.10G007820.1	0.4214	2.4365	0.1729	Segmental
Soltu.DM.02G026200.1	Soltu.DM.03G004620.1	0.4258	1.0359	0.4110	Segmental
Soltu.DM.02G026200.1	Soltu.DM.03G033630.1	0.4918	3.6522	0.1346	Segmental
Soltu.DM.03G033630.1	Soltu.DM.06G025000.1	0.1427	0.6020	0.2370	Segmental
Soltu.DM.03G000250.1	Soltu.DM.06G015080.1	0.2540	0.6420	0.3957	Segmental
Soltu.DM.03G000280.1	Soltu.DM.06G015050.1	0.4943	1.1736	0.4211	Segmental
Soltu.DM.03G000250.1	Soltu.DM.09G024650.1	0.1960	0.7479	0.2620	Segmental
Soltu.DM.03G000280.1	Soltu.DM.09G024670.1	0.3285	0.8328	0.3944	Segmental
Soltu.DM.06G017790.1	Soltu.DM.08G025180.1	0.2046	0.6983	0.2931	Segmental
Soltu.DM.06G034620.1	Soltu.DM.09G020000.2	0.4151	0.9806	0.4233	Segmental
Soltu.DM.06G034630.1	Soltu.DM.09G019990.1	0.2520	0.8660	0.2910	Segmental
Soltu.DM.06G015050.1	Soltu.DM.09G024670.1	0.5450	2.0130	0.2708	Segmental
Soltu.DM.06G015070.1	Soltu.DM.09G024650.1	0.5525	2.6569	0.2079	Segmental
Soltu.DM.06G015050.1	Soltu.DM.09G020020.1	0.5489	1.2173	0.4509	Segmental
Soltu.DM.06G015070.1	Soltu.DM.09G020000.2	0.4436	1.3691	0.3240	Segmental
Soltu.DM.07G013810.1	Soltu.DM.12G026740.1	0.4234	1.3943	0.3037	Segmental
Soltu.DM.08G002860.1	Soltu.DM.08G018950.1	0.1098	0.3732	0.2941	Segmental
Soltu.DM.08G000480.1	Soltu.DM.08G025530.1	0.3035	1.0604	0.2862	Segmental
Soltu.DM.09G019990.1	Soltu.DM.09G024650.1	0.5236	1.1720	0.4468	Segmental
Soltu.DM.09G020020.1	Soltu.DM.09G024670.1	0.5595	1.3569	0.4124	Segmental
Soltu.DM.01G028720.1	Soltu.DM.01G028730.1	0.7530	1.6029	0.4698	Tandem
Soltu.DM.01G031010.1	Soltu.DM.01G031020.1	0.1064	0.3443	0.3089	Tandem
Soltu.DM.03G004620.1	Soltu.DM.03G004630.1	0.2420	0.6181	0.3916	Tandem
Soltu.DM.03G004670.1	Soltu.DM.03G004690.1	0.4497	0.5659	0.7946	Tandem
Soltu.DM.03G015130.1	Soltu.DM.03G015150.1	0.1900	0.2152	0.8832	Tandem
Soltu.DM.03G015150.1	Soltu.DM.03G015160.1	0.1364	0.4403	0.3098	Tandem
Soltu.DM.06G003700.1	Soltu.DM.06G003710.1	0.3251	0.6617	0.4913	Tandem
Soltu.DM.06G003710.1	Soltu.DM.06G003720.1	0.3106	0.6553	0.4740	Tandem
Soltu.DM.06G003720.1	Soltu.DM.06G003730.1	0.3017	0.8690	0.3472	Tandem
Soltu.DM.06G015070.1	Soltu.DM.06G015080.1	0.5685	3.0102	0.1889	Tandem
Soltu.DM.06G016150.1	Soltu.DM.06G016170.1	0.1683	0.2801	0.6007	Tandem
Soltu.DM.06G034620.1	Soltu.DM.06G034630.1	0.5390	1.7621	0.3059	Tandem
Soltu.DM.08G016080.1	Soltu.DM.08G016090.1	0.5226	1.4369	0.3637	Tandem
Soltu.DM.08G016090.1	Soltu.DM.08G016100.1	0.4869	1.7406	0.2797	Tandem
Soltu.DM.08G016140.1	Soltu.DM.08G016150.1	0.2797	0.4623	0.6050	Tandem
Soltu.DM.08G016150.1	Soltu.DM.08G016160.1	0.3713	0.3966	0.9364	Tandem
Soltu.DM.08G023700.1	Soltu.DM.08G023710.1	0.1131	0.1626	0.6955	Tandem
Soltu.DM.08G025200.1	Soltu.DM.08G025210.1	0.5592	1.9142	0.2921	Tandem
Soltu.DM.08G025210.1	Soltu.DM.08G025220.1	0.2529	0.9422	0.2684	Tandem
Soltu.DM.08G025220.1	Soltu.DM.08G025230.1	0.3007	2.1893	0.1373	Tandem
Soltu.DM.08G025240.1	Soltu.DM.08G025250.1	0.3220	1.3326	0.2416	Tandem

Soltu.DM.08G025480.1	Soltu.DM.08G025490.1	0.0392	0.1156	0.3389	Tandem
Soltu.DM.08G025490.1	Soltu.DM.08G025500.1	0.0394	0.1056	0.3732	Tandem
Soltu.DM.08G025530.1	Soltu.DM.08G025540.1	0.1683	0.2803	0.6002	Tandem
Soltu.DM.08G027130.1	Soltu.DM.08G027140.1	0.3822	1.8306	0.2088	Tandem
Soltu.DM.09G020000.2	Soltu.DM.09G020010.1	0.4079	1.1098	0.3675	Tandem
Soltu.DM.09G020010.1	Soltu.DM.09G020020.1	0.7648	1.5087	0.5069	Tandem
Soltu.DM.09G024670.1	Soltu.DM.09G024680.1	0.5753	1.3499	0.4262	Tandem
Soltu.DM.10G006020.1	Soltu.DM.10G006030.1	0.1186	0.0906	1.3089	Tandem
Soltu.DM.10G006030.1	Soltu.DM.10G006050.1	0.1188	0.1048	1.1337	Tandem
Soltu.DM.10G006050.1	Soltu.DM.10G006060.1	0.2025	0.1201	1.6857	Tandem
Soltu.DM.10G006060.1	Soltu.DM.10G006090.1	0.1606	0.0400	4.0129	Tandem
Soltu.DM.10G018660.1	Soltu.DM.10G018670.1	0.1840	0.6460	0.2849	Tandem
Soltu.DM.10G018670.1	Soltu.DM.10G018680.1	0.1528	0.3938	0.3881	Tandem
Soltu.DM.10G018680.1	Soltu.DM.10G018690.1	0.0659	0.4115	0.1602	Tandem
Soltu.DM.10G018690.1	Soltu.DM.10G018700.1	0.0615	0.1786	0.3445	Tandem
Soltu.DM.10G018740.1	Soltu.DM.10G018750.1	0.0502	0.2229	0.2254	Tandem
Soltu.DM.10G018750.1	Soltu.DM.10G018760.1	0.0352	0.1025	0.3434	Tandem
Soltu.DM.10G018760.1	Soltu.DM.10G018770.1	0.0081	0.1087	0.0748	Tandem
Soltu.DM.10G018770.1	Soltu.DM.10G018780.1	0.0081	0.0963	0.0843	Tandem
Soltu.DM.10G018780.1	Soltu.DM.10G018790.1	0.0593	0.3145	0.1885	Tandem
Soltu.DM.10G018790.1	Soltu.DM.10G018810.1	0.0079	0.0354	0.2228	Tandem
Soltu.DM.10G018820.1	Soltu.DM.10G018830.1	0.1558	0.5367	0.2902	Tandem
Soltu.DM.10G018830.1	Soltu.DM.10G018840.1	0.1292	0.4813	0.2683	Tandem
Soltu.DM.10G018860.1	Soltu.DM.10G018870.1	0.3252	0.6685	0.4864	Tandem
Soltu.DM.10G018660.1	Nitab4.5_0000046g0200.1	0.4371	1.8789	0.2326	St-Nt
Soltu.DM.10G018860.1	Nitab4.5_0002009g0010.1	0.3123	0.6150	0.5078	St-Nt
Soltu.DM.10G018660.1	Nitab4.5_0002204g0030.1	0.3000	0.6209	0.4832	St-Nt
Soltu.DM.11G025020.1	Nitab4.5_0002567g0050.1	0.2102	0.6514	0.3227	St-Nt
Soltu.DM.12G026740.1	Nitab4.5_0000170g0310.1	0.4176	1.2127	0.3444	St-Nt
Soltu.DM.01G001550.1	Nitab4.5_0000775g0090.1	0.1226	0.3685	0.3326	St-Nt
Soltu.DM.01G042060.1	Nitab4.5_0003120g0010.1	0.2129	1.0692	0.1991	St-Nt
Soltu.DM.01G021910.1	Nitab4.5_0000960g0110.1	0.0909	0.2815	0.3230	St-Nt
Soltu.DM.01G031610.1	Nitab4.5_0000859g0300.1	0.0836	0.3048	0.2742	St-Nt
Soltu.DM.01G031010.1	Nitab4.5_0003696g0040.1	0.2717	2.1037	0.1292	St-Nt
Soltu.DM.01G028720.1	Nitab4.5_0000468g0250.1	0.1903	0.3344	0.5692	St-Nt
Soltu.DM.01G028630.1	Nitab4.5_0000468g0150.1	0.0662	0.2282	0.2903	St-Nt
Soltu.DM.01G027870.1	Nitab4.5_0001542g0080.1	0.1242	0.2498	0.4971	St-Nt
Soltu.DM.01G042060.1	Nitab4.5_0004668g0080.1	0.2561	0.8706	0.2942	St-Nt
Soltu.DM.01G031010.1	Nitab4.5_0000851g0010.1	0.1969	1.2333	0.1597	St-Nt
Soltu.DM.02G031780.1	Nitab4.5_0006318g0080.1	0.1614	0.6779	0.2381	St-Nt
Soltu.DM.02G026200.1	Nitab4.5_0000625g0070.1	0.5758	0.9386	0.6134	St-Nt
Soltu.DM.02G026200.1	Nitab4.5_0000965g0060.1	0.3410	1.1175	0.3052	St-Nt
Soltu.DM.02G026200.1	Nitab4.5_0004279g0030.1	0.3559	0.8591	0.4143	St-Nt
Soltu.DM.02G031780.1	Nitab4.5_0000564g0110.1	0.1496	0.5681	0.2633	St-Nt

Soltu.DM.07G013810.1	Nitab4.5_0000170g0310.1	0.0954	0.4849	0.1968	St-Nt
Soltu.DM.07G026820.1	Nitab4.5_0001160g0120.1	0.2160	0.5780	0.3737	St-Nt
Soltu.DM.07G020060.1	Nitab4.5_0000638g0010.1	0.2638	0.6749	0.3909	St-Nt
Soltu.DM.07G025650.1	Nitab4.5_0000397g0180.1	0.2087	0.6170	0.3382	St-Nt
Soltu.DM.08G025480.1	Nitab4.5_0003983g0030.1	0.0647	0.2965	0.2181	St-Nt
Soltu.DM.08G025250.1	Nitab4.5_0004430g0090.1	0.1144	0.5402	0.2117	St-Nt
Soltu.DM.08G025220.1	Nitab4.5_0004430g0100.1	0.0715	0.3940	0.1815	St-Nt
Soltu.DM.08G025180.1	Nitab4.5_0004430g0020.1	0.2938	2.6535	0.1107	St-Nt
Soltu.DM.08G016080.1	Nitab4.5_0002140g0020.1	0.5755	1.6925	0.3400	St-Nt
Soltu.DM.08G025200.1	Nitab4.5_0000558g0010.1	0.5395	2.4378	0.2213	St-Nt
Soltu.DM.08G000480.1	Nitab4.5_0001816g0220.1	0.1314	0.6302	0.2085	St-Nt
Soltu.DM.08G002860.1	Nitab4.5_0000860g0060.1	0.0612	0.3586	0.1705	St-Nt
Soltu.DM.08G018950.1	Nitab4.5_0000860g0060.1	0.1139	0.4213	0.2703	St-Nt
Soltu.DM.08G023700.1	Nitab4.5_0000185g0190.1	0.3010	1.1161	0.2697	St-Nt
Soltu.DM.08G016090.1	Nitab4.5_0000125g0010.1	0.0963	0.4997	0.1927	St-Nt
Soltu.DM.08G025180.1	Nitab4.5_0000312g0240.1	0.0584	0.6114	0.0956	St-Nt
Soltu.DM.08G025200.1	Nitab4.5_0000312g0280.1	0.0740	0.4947	0.1496	St-Nt
Soltu.DM.08G025250.1	Nitab4.5_0000312g0320.1	0.0827	0.5846	0.1414	St-Nt
Soltu.DM.08G025480.1	Nitab4.5_0004482g0070.1	0.1922	0.6063	0.3171	St-Nt
Soltu.DM.08G025530.1	Nitab4.5_0004482g0010.1	0.2099	0.4727	0.4439	St-Nt
Soltu.DM.08G000480.1	Nitab4.5_0004482g0010.1	0.2283	1.0397	0.2195	St-Nt
Soltu.DM.08G016080.1	Nitab4.5_0000508g0230.1	0.2466	0.6652	0.3707	St-Nt
Soltu.DM.09G010750.1	Nitab4.5_0000046g0200.1	0.4332	2.3480	0.1845	St-Nt
Soltu.DM.09G010750.1	Nitab4.5_0003323g0030.1	0.1731	0.5303	0.3264	St-Nt
Soltu.DM.09G024650.1	Nitab4.5_0001157g0050.1	0.1051	0.4546	0.2311	St-Nt
Soltu.DM.09G024670.1	Nitab4.5_0001157g0060.1	0.1709	0.6005	0.2845	St-Nt
Soltu.DM.09G024680.1	Nitab4.5_0001157g0080.1	0.0949	0.3726	0.2547	St-Nt
Soltu.DM.09G024690.1	Nitab4.5_0002307g0080.1	0.2131	0.4131	0.5158	St-Nt
Soltu.DM.09G019990.1	Nitab4.5_0001157g0050.1	0.6242	2.0987	0.2974	St-Nt
Soltu.DM.09G020000.2	Nitab4.5_0001157g0040.1	0.4194	1.4941	0.2807	St-Nt
Soltu.DM.09G020020.1	Nitab4.5_0001157g0060.1	0.5870	1.7044	0.3444	St-Nt
Soltu.DM.09G020020.1	Nitab4.5_0000252g0010.1	0.1452	0.4185	0.3471	St-Nt
Soltu.DM.09G019990.1	Nitab4.5_0000252g0030.1	0.2735	1.0325	0.2649	St-Nt
Soltu.DM.09G024670.1	Nitab4.5_0000252g0010.1	0.5807	1.4300	0.4061	St-Nt
Soltu.DM.09G024650.1	Nitab4.5_0000252g0030.1	0.4929	1.3548	0.3638	St-Nt
Soltu.DM.03G017060.1	Nitab4.5_0002107g0020.1	0.0991	0.1775	0.5584	St-Nt
Soltu.DM.03G015150.1	Nitab4.5_0000558g0050.1	0.1296	0.6589	0.1967	St-Nt
Soltu.DM.03G012340.1	Nitab4.5_0002822g0020.1	0.0971	0.8132	0.1195	St-Nt
Soltu.DM.03G032810.1	Nitab4.5_0002046g0010.1	0.2193	0.9803	0.2237	St-Nt
Soltu.DM.03G034110.1	Nitab4.5_0001599g0040.1	0.1011	0.3098	0.3262	St-Nt
Soltu.DM.03G017060.1	Nitab4.5_0004843g0030.1	0.1198	0.3202	0.3741	St-Nt
Soltu.DM.03G004620.1	Nitab4.5_0000965g0060.1	0.4463	1.0928	0.4084	St-Nt
Soltu.DM.03G004620.1	Nitab4.5_0004136g0010.1	0.2018	0.5076	0.3975	St-Nt
Soltu.DM.05G011870.1	Nitab4.5_0004080g0010.1	0.1228	0.6177	0.1988	St-Nt

Soltu.DM.06G025000.1	Nitab4.5_0001194g0100.1	0.0551	0.3475	0.1586	St-Nt
Soltu.DM.06G029040.1	Nitab4.5_0002184g0120.1	0.1316	0.2840	0.4631	St-Nt
Soltu.DM.06G003700.1	Nitab4.5_0000102g0410.1	0.2582	0.7415	0.3482	St-Nt
Soltu.DM.06G034620.1	Nitab4.5_0000252g0030.1	0.2499	0.6130	0.4076	St-Nt
Soltu.DM.06G015050.1	Nitab4.5_0000252g0010.1	0.5644	1.5873	0.3556	St-Nt
Soltu.DM.06G015070.1	Nitab4.5_0000252g0030.1	0.3648	2.5050	0.1456	St-Nt
Soltu.DM.06G017790.1	Nitab4.5_0000312g0240.1	0.1745	0.5686	0.3069	St-Nt
Soltu.DM.10G007820.1	Solyc01g090350.3.1	0.4454	2.0598	0.2162	St-Sl
Soltu.DM.10G006020.1	Solyc10g012110.3.1	0.1738	0.2233	0.7783	St-Sl
Soltu.DM.10G018660.1	Solyc10g075050.2.1	0.0645	0.2465	0.2615	St-Sl
Soltu.DM.10G007820.1	Solyc10g039197.2.1	0.0372	0.1244	0.2990	St-Sl
Soltu.DM.11G025020.1	Solyc11g072030.3.1	0.0439	0.1003	0.4380	St-Sl
Soltu.DM.12G026740.1	Solyc07g007860.1.1	0.1588	0.8168	0.1944	St-Sl
Soltu.DM.12G026740.1	Solyc12g009650.2.1	0.0804	0.2245	0.3583	St-Sl
Soltu.DM.01G031010.1	Solyc01g090350.3.1	0.0429	0.1333	0.3217	St-Sl
Soltu.DM.01G031610.1	Solyc01g090965.1.1	0.0349	0.1727	0.2021	St-Sl
Soltu.DM.01G034890.1	Solyc01g095780.2.1	0.0575	0.1315	0.4370	St-Sl
Soltu.DM.01G042060.1	Solyc01g103060.4.1	0.0472	0.1166	0.4051	St-Sl
Soltu.DM.01G049040.1	Solyc01g109390.3.1	0.0382	0.0440	0.8671	St-Sl
Soltu.DM.01G001550.1	Solyc01g005985.1.1	0.0325	0.1447	0.2242	St-Sl
Soltu.DM.01G021910.1	Solyc01g066910.3.1	0.0449	0.1665	0.2698	St-Sl
Soltu.DM.01G028730.1	Solyc01g081590.4.1	0.0535	0.0584	0.9166	St-Sl
Soltu.DM.01G028720.1	Solyc01g081600.3.1	0.0318	0.1402	0.2267	St-Sl
Soltu.DM.01G028630.1	Solyc01g073680.4.1	0.0231	0.0831	0.2785	St-Sl
Soltu.DM.01G027870.1	Solyc01g086840.3.1	0.0480	0.0913	0.5255	St-Sl
Soltu.DM.01G031610.1	Solyc08g078870.3.1	0.3613	2.2948	0.1574	St-Sl
Soltu.DM.01G031610.1	Solyc08g079200.1.1	0.3909	1.6012	0.2441	St-Sl
Soltu.DM.02G026200.1	Solyc02g086310.2.1	0.3935	0.4451	0.8839	St-Sl
Soltu.DM.02G031780.1	Solyc02g087910.3.1	0.0605	0.1216	0.4975	St-Sl
Soltu.DM.02G026200.1	Solyc03g034330.1.1	0.4903	1.1681	0.4197	St-Sl
Soltu.DM.07G025650.1	Solyc07g064210.2.1	0.0351	0.2028	0.1730	St-Sl
Soltu.DM.07G026820.1	Solyc07g065110.1.1	0.0780	0.1501	0.5198	St-Sl
Soltu.DM.07G020060.1	Solyc10g008205.2.1	0.3960	1.1993	0.3302	St-Sl
Soltu.DM.07G013810.1	Solyc12g009650.2.1	0.4443	1.2808	0.3469	St-Sl
Soltu.DM.08G025200.1	Solyc03g083990.1.1	0.6603	1.6590	0.3980	St-Sl
Soltu.DM.08G025180.1	Solyc06g060640.1.1	0.2211	0.7633	0.2897	St-Sl
Soltu.DM.08G016080.1	Solyc08g067490.1.1	0.1730	0.2144	0.8067	St-Sl
Soltu.DM.08G016090.1	Solyc08g067510.1.1	0.0296	0.0819	0.3614	St-Sl
Soltu.DM.08G018950.1	Solyc08g074480.1.1	0.0172	0.1078	0.1599	St-Sl
Soltu.DM.08G025180.1	Solyc08g078870.3.1	0.0169	0.1024	0.1654	St-Sl
Soltu.DM.08G025200.1	Solyc08g078890.3.1	0.0211	0.1429	0.1474	St-Sl
Soltu.DM.08G025480.1	Solyc08g079200.1.1	0.0541	0.1209	0.4475	St-Sl
Soltu.DM.08G025530.1	Solyc08g079250.4.1	0.1876	0.2494	0.7523	St-Sl
Soltu.DM.08G002860.1	Solyc08g074480.1.1	0.1042	0.4255	0.2449	St-Sl

Soltu.DM.08G025530.1	Solyc08g007460.3.1	0.3042	1.1591	0.2625	St-Sl
Soltu.DM.08G002860.1	Solyc08g005960.2.1	0.0099	0.0821	0.1205	St-Sl
Soltu.DM.08G000480.1	Solyc08g007460.3.1	0.0393	0.1177	0.3336	St-Sl
Soltu.DM.08G000480.1	Solyc08g079250.4.1	0.3463	1.1183	0.3097	St-Sl
Soltu.DM.08G018950.1	Solyc08g005960.2.1	0.1176	0.4067	0.2890	St-Sl
Soltu.DM.09G019990.1	Solyc06g084190.3.1	0.2864	1.0385	0.2758	St-Sl
Soltu.DM.09G024670.1	Solyc06g054030.2.1	0.5367	2.2086	0.2430	St-Sl
Soltu.DM.09G024650.1	Solyc06g054060.1.1	0.5858	2.2364	0.2620	St-Sl
Soltu.DM.09G020020.1	Solyc06g054030.2.1	0.5374	1.5078	0.3564	St-Sl
Soltu.DM.09G020000.2	Solyc06g054070.3.1	0.5263	1.8127	0.2903	St-Sl
Soltu.DM.09G019990.1	Solyc09g065410.4.1	0.0749	0.1516	0.4938	St-Sl
Soltu.DM.09G020020.1	Solyc09g065440.4.1	0.0340	0.1283	0.2652	St-Sl
Soltu.DM.09G020300.1	Solyc09g150148.1.1	0.0066	0.1312	0.0504	St-Sl
Soltu.DM.09G024650.1	Solyc09g082270.4.1	0.0458	0.1612	0.2842	St-Sl
Soltu.DM.09G024670.1	Solyc09g082290.3.1	0.0668	0.1198	0.5580	St-Sl
Soltu.DM.09G024690.1	Solyc09g082310.2.1	0.0490	0.1218	0.4021	St-Sl
Soltu.DM.09G019990.1	Solyc09g082270.4.1	0.5746	1.1910	0.4824	St-Sl
Soltu.DM.09G020000.2	Solyc09g082280.3.1	0.3419	1.4519	0.2355	St-Sl
Soltu.DM.09G020020.1	Solyc09g082290.3.1	0.5354	1.5600	0.3432	St-Sl
Soltu.DM.09G024650.1	Solyc09g065410.4.1	0.5382	1.1947	0.4505	St-Sl
Soltu.DM.09G024670.1	Solyc09g065440.4.1	0.5873	1.4956	0.3927	St-Sl
Soltu.DM.09G005000.1	Solyc09g008500.3.1	0.2739	0.2196	1.2474	St-Sl
Soltu.DM.09G010750.1	Solyc09g018010.3.1	0.1881	0.0877	2.1457	St-Sl
Soltu.DM.09G010750.1	Solyc10g075050.2.1	0.2731	1.6597	0.1645	St-Sl
Soltu.DM.03G032810.1	Solyc03g118580.3.1	0.1414	0.2511	0.5632	St-Sl
Soltu.DM.03G033630.1	Solyc03g119210.1.1	0.0347	0.1352	0.2569	St-Sl
Soltu.DM.03G034110.1	Solyc03g119620.1.1	0.0346	0.1314	0.2633	St-Sl
Soltu.DM.03G036710.1	Solyc03g121900.1.1	0.0296	0.2098	0.1409	St-Sl
Soltu.DM.03G004560.1	Solyc03g034330.1.1	0.0673	0.2615	0.2572	St-Sl
Soltu.DM.03G004630.1	Solyc03g034390.1.1	0.3646	0.6414	0.5684	St-Sl
Soltu.DM.03G000250.1	Solyc03g005200.3.1	0.0391	0.1281	0.3051	St-Sl
Soltu.DM.03G000280.1	Solyc03g005210.4.1	0.0485	0.0659	0.7367	St-Sl
Soltu.DM.03G012340.1	Solyc03g079880.4.1	0.0040	0.0902	0.0438	St-Sl
Soltu.DM.03G004620.1	Solyc03g034330.1.1	0.0697	0.2946	0.2366	St-Sl
Soltu.DM.03G015160.1	Solyc03g083996.1.1	0.0348	0.2296	0.1515	St-Sl
Soltu.DM.03G015150.1	Solyc03g090990.1.1	0.1814	0.1940	0.9351	St-Sl
Soltu.DM.03G017060.1	Solyc03g026090.4.1	0.0215	0.1337	0.1605	St-Sl
Soltu.DM.03G033630.1	Solyc05g008895.1.1	0.5169	2.0705	0.2497	St-Sl
Soltu.DM.03G033630.1	Solyc06g069070.1.1	0.1793	0.5219	0.3436	St-Sl
Soltu.DM.03G000280.1	Solyc06g054030.2.1	0.5397	1.3631	0.3959	St-Sl
Soltu.DM.03G000250.1	Solyc06g054060.1.1	0.5471	2.6992	0.2027	St-Sl
Soltu.DM.03G015150.1	Solyc08g078900.1.1	0.2803	2.9568	0.0948	St-Sl
Soltu.DM.03G000250.1	Solyc09g082270.4.1	0.2044	0.8420	0.2427	St-Sl
Soltu.DM.03G000280.1	Solyc09g082290.3.1	0.3445	0.8434	0.4085	St-Sl

Soltu.DM.05G011870.1	Solyc05g015490.4.1	0.0378	0.2818	0.1340	St-Sl
Soltu.DM.06G015070.1	Solyc03g005200.3.1	0.5568	1.9155	0.2907	St-Sl
Soltu.DM.06G015050.1	Solyc03g005210.4.1	0.5466	1.2082	0.4525	St-Sl
Soltu.DM.06G025000.1	Solyc06g069070.1.1	0.0290	0.1381	0.2102	St-Sl
Soltu.DM.06G025060.1	Solyc06g069110.4.1	0.0221	0.1376	0.1608	St-Sl
Soltu.DM.06G029040.1	Solyc06g073660.3.1	0.0481	0.1063	0.4520	St-Sl
Soltu.DM.06G034620.1	Solyc06g084190.3.1	0.5396	1.8362	0.2938	St-Sl
Soltu.DM.06G015050.1	Solyc06g054030.2.1	0.0466	0.0722	0.6453	St-Sl
Soltu.DM.06G015070.1	Solyc06g054060.1.1	0.0418	0.0929	0.4495	St-Sl
Soltu.DM.06G015080.1	Solyc06g054070.3.1	0.0753	0.0564	1.3350	St-Sl
Soltu.DM.06G016150.1	Solyc06g059790.4.1	0.0218	0.1521	0.1433	St-Sl
Soltu.DM.06G017790.1	Solyc06g060640.1.1	0.0132	0.0647	0.2045	St-Sl
Soltu.DM.06G013400.1	Solyc06g036230.4.1	0.2816	0.2827	0.9963	St-Sl
Soltu.DM.06G034620.1	Solyc06g054060.1.1	0.4269	1.5457	0.2762	St-Sl
Soltu.DM.06G003700.1	Solyc06g005770.1.1	0.3236	0.6925	0.4673	St-Sl
Soltu.DM.06G019440.1	Solyc06g065970.1.1	0.0253	0.0648	0.3906	St-Sl
Soltu.DM.06G017790.1	Solyc08g078870.3.1	0.1974	0.6308	0.3130	St-Sl
Soltu.DM.06G034620.1	Solyc09g065410.4.1	0.5810	1.7308	0.3357	St-Sl
Soltu.DM.06G015050.1	Solyc09g082290.3.1	0.4972	1.1887	0.4183	St-Sl
Soltu.DM.06G015070.1	Solyc09g065410.4.1	0.7848	2.0252	0.3875	St-Sl
Soltu.DM.06G015050.1	Solyc09g065440.4.1	0.5378	1.2044	0.4465	St-Sl
Soltu.DM.09G010750.1	Os11t0114900-01	0.5061	2.9445	0.1719	St-Os
Soltu.DM.09G010750.1	Os12t0114500-01	0.4824	3.6804	0.1311	St-Os
Soltu.DM.03G033630.1	Os05t0550300-01	0.5080	2.8125	0.1806	St-Os
Soltu.DM.07G013810.1	AT3G22120.2	0.2626	2.2176	0.1184	St-At
Soltu.DM.07G013810.1	AT4G15160.1	0.2802	1.7812	0.1573	St-At
Soltu.DM.08G025200.1	AT1G62500.1	0.2977	1.8024	0.1652	St-At
Soltu.DM.08G025540.1	AT1G62790.1	0.5672	2.1744	0.2609	St-At
Soltu.DM.08G025180.1	AT2G45180.1	0.3212	1.9318	0.1663	St-At
Soltu.DM.08G025210.1	AT4G22460.1	0.3433	1.8287	0.1877	St-At
Soltu.DM.08G025200.1	AT4G12470.1	0.6159	1.9446	0.3167	St-At
Soltu.DM.09G010750.1	AT2G38530.1	0.4911	2.2144	0.2218	St-At
Soltu.DM.09G024650.1	AT3G22600.1	0.4569	2.1399	0.2135	St-At
Soltu.DM.09G024670.1	AT3G22620.1	0.5519	2.1189	0.2605	St-At
Soltu.DM.09G019990.1	AT3G22600.1	0.5925	1.4968	0.3959	St-At
Soltu.DM.09G020020.1	AT3G22620.1	0.5937	2.3682	0.2507	St-At
Soltu.DM.09G024690.1	AT4G14805.1	0.5700	1.5604	0.3653	St-At
Soltu.DM.09G024650.1	AT4G14815.1	0.5078	2.1785	0.2331	St-At
Soltu.DM.09G019990.1	AT4G14815.1	0.6307	1.7723	0.3559	St-At
Soltu.DM.09G010750.1	AT5G01870.1	0.6557	1.1749	0.5580	St-At
Soltu.DM.03G004690.1	AT1G66850.1	0.6383	2.0478	0.3117	St-At
Soltu.DM.03G033630.1	AT3G18280.2	0.5364	2.6362	0.2035	St-At
Soltu.DM.03G000250.1	AT3G22600.1	0.4341	2.8060	0.1547	St-At
Soltu.DM.03G000280.1	AT3G22620.1	0.4940	2.1053	0.2347	St-At

Soltu.DM.03G000250.1	AT4G14815.1	0.5323	2.4477	0.2175	St-At
Soltu.DM.03G017060.1	AT5G62065.1	0.4445	3.2868	0.1352	St-At
Soltu.DM.05G011870.1	AT1G27950.1	0.5270	3.6011	0.1464	St-At
Soltu.DM.06G034620.1	AT1G03103.1	0.5403	2.5495	0.2119	St-At
Soltu.DM.06G017790.1	AT2G45180.1	0.2967	1.6613	0.1786	St-At
Soltu.DM.06G015050.1	AT3G22620.1	0.5548	1.7722	0.3130	St-At
Soltu.DM.06G015070.1	AT4G14815.1	0.6670	1.9529	0.3415	St-At

Note: Non-synonymous substitution rate (Ka) and synonymous substitution rate (Ks) of duplicated and orthologous gene pairs were calculated using the Simple Ka/Ks Calculator function module of the TBtools software. Ka/Ks < 1 indicates that the gene pairs have undergone purifying selection during evolution.

St-Nt: *Solanum tuberosum* (potato) – *Nicotiana tabacum* (tobacco) orthologous gene pairs;

St-Sl: *Solanum tuberosum* (potato) – *Solanum lycopersicum* (tomato) orthologous gene pairs;

St-Os: *Solanum tuberosum* (potato) – *Oryza sativa* (rice) orthologous gene pairs;

St-At: *Solanum tuberosum* (potato) – *Arabidopsis thaliana* (Arabidopsis) orthologous gene pairs;

Segmental: Segmental duplication gene pairs in potato;

Tandem: Tandem duplication gene pairs in potato.
