# Supplementary material for: Genome-Wide Characterization and Expression Analysis of the nsLTP Gene Family in Potato (Solanum tuberosum) in Response to Drought Stress
Source: Biology (Basel). 2026 Oct 28;15(21):1947. doi: 10.3390/biology15211947 (PMC13761951; doi:10.3390/biology15211947)
Supplement: Supplementary file 1 [file biology-15-01947-s001.zip › Supplementary Table/Supplementary Table S6.pdf]

Supplementary Table S6 Gene Ontology (GO) enrichment analysis of potato *StnsLTP* family genes

Class	GO Name	GO ID	GO Level	P-value	Enrichment Score	HitsInSet	HitsInBG	AllInSet	AllInBG
Molecular function	binding	GO:0005488	2	0.00797	2.24	6	4914	6	10991
Cellular component	anchored component of membrane	GO:0031225	0	2.08E-09	28.70	7	173	18	12768
	anchored component of plasma membrane	GO:0046658	0	1.91E-08	32.00	6	133	18	12768
	membrane	GO:0016020	3	9.56E-07	2.46	17	4899	18	12768
Biological process	lipid transport	GO:0006869	5	0	94.55	13	111	16	12917
	lipid localization	GO:0010876	4	0	72.38	13	145	16	12917
	macromolecule localization	GO:0033036	3	8.03E-14	13.17	13	797	16	12917

Note: GO enrichment analysis of *StnsLTP* genes was conducted using the TBtools software with default parameters, based on the potato genome annotation.

Abbreviations: HitsInSet, number of genes in the input list annotated to the GO term; HitsInBG, number of genes in the background annotated to the GO term; AllInSet, total number of genes in the input list; AllInBG, total number of genes in the background.
