# Supplementary material for: Genome-Wide Characterization and Expression Analysis of the nsLTP Gene Family in Potato (Solanum tuberosum) in Response to Drought Stress
Source: Biology (Basel). 2026 Oct 28;15(21):1947. doi: 10.3390/biology15211947 (PMC13761951; doi:10.3390/biology15211947)
Supplement: Supplementary file 1 [file biology-15-01947-s001.zip › Supplementary Table/Supplementary Table S7.pdf]

Supplementary Table S7 Correspondence between *nsLTP* genes identified in the previous study (DM v4.03 genome) and *nsLTP* genes identified in this study (DM v6.1 genome)

PGSC DM v4.03 Gene ID	DM v6.1 Gene Locus ID	Gene name	PGSC DM v4.03 Gene ID	DM v6.1 Gene Locus ID	Gene name
PGSC0003DMG400026381	Soltu.DM.02G026200.1	StnsLTP.013	PGSC0003DMG400019325	Soltu.DM.06G015070.1	StnsLTP.044
PGSC0003DMG400001322	Soltu.DM.02G031780.1	StnsLTP.014	PGSC0003DMG400019325	Soltu.DM.09G019990.1	StnsLTP.087
PGSC0003DMG400026381	Soltu.DM.03G004620.1	StnsLTP.018	PGSC0003DMG400032250	Soltu.DM.09G024650.1	StnsLTP.092
PGSC0003DMG400026381	Soltu.DM.03G004560.1	StnsLTP.017	PGSC0003DMG400032260	Soltu.DM.09G024670.1	StnsLTP.094
PGSC0003DMG400026381	Soltu.DM.03G033630.1	StnsLTP.033	PGSC0003DMG400001904	Soltu.DM.09G010750.1	StnsLTP.086
PGSC0003DMG400011323	Soltu.DM.01G021910.1	StnsLTP.002	PGSC0003DMG400032250	Soltu.DM.09G019990.1	StnsLTP.087
PGSC0003DMG400028977	Soltu.DM.01G028630.1	StnsLTP.004	PGSC0003DMG400032260	Soltu.DM.09G020020.1	StnsLTP.090
PGSC0003DMG400031126	Soltu.DM.01G028730.1	StnsLTP.006	PGSC0003DMG400019325	Soltu.DM.09G024650.1	StnsLTP.092
PGSC0003DMG400025988	Soltu.DM.01G031010.1	StnsLTP.007	PGSC0003DMG402016309	Soltu.DM.03G000250.1	StnsLTP.015
PGSC0003DMG400000218	Soltu.DM.01G034890.1	StnsLTP.010	PGSC0003DMG402020132	Soltu.DM.03G000250.1	StnsLTP.015
PGSC0003DMG400020710	Soltu.DM.01G042060.1	StnsLTP.011	PGSC0003DMG400028909	Soltu.DM.06G013400.1	StnsLTP.042
PGSC0003DMG400025168	Soltu.DM.01G049040.1	StnsLTP.012	PGSC0003DMG402016309	Soltu.DM.06G015070.1	StnsLTP.044
PGSC0003DMG400032182	Soltu.DM.01G001550.1	StnsLTP.001	PGSC0003DMG401016309	Soltu.DM.06G015080.1	StnsLTP.045
PGSC0003DMG400025988	Soltu.DM.10G007820.1	StnsLTP.102	PGSC0003DMG400030768	Soltu.DM.06G016150.1	StnsLTP.046
PGSC0003DMG400021763	Soltu.DM.02G026200.1	StnsLTP.013	PGSC0003DMG400030768	Soltu.DM.06G025060.1	StnsLTP.051
PGSC0003DMG400031724	Soltu.DM.03G032810.1	StnsLTP.031	PGSC0003DMG402020132	Soltu.DM.06G034620.1	StnsLTP.053
PGSC0003DMG400005731	Soltu.DM.03G033630.1	StnsLTP.033	PGSC0003DMG400020823	Soltu.DM.06G003700.1	StnsLTP.038
PGSC0003DMG400002487	Soltu.DM.03G036710.1	StnsLTP.035	PGSC0003DMG402020132	Soltu.DM.06G015070.1	StnsLTP.044
PGSC0003DMG400013434	Soltu.DM.03G000250.1	StnsLTP.015	PGSC0003DMG402016309	Soltu.DM.06G034620.1	StnsLTP.053
PGSC0003DMG400021763	Soltu.DM.03G004620.1	StnsLTP.018	PGSC0003DMG402020132	Soltu.DM.09G019990.1	StnsLTP.087
PGSC0003DMG400021761	Soltu.DM.03G004670.1	StnsLTP.020	PGSC0003DMG402016309	Soltu.DM.09G024650.1	StnsLTP.092
PGSC0003DMG400021691	Soltu.DM.03G012340.1	StnsLTP.022	PGSC0003DMG401016309	Soltu.DM.09G020000.2	StnsLTP.088
PGSC0003DMG400021773	Soltu.DM.03G004630.1	StnsLTP.019	PGSC0003DMG400030587	Soltu.DM.03G033630.1	StnsLTP.033
PGSC0003DMG400013434	Soltu.DM.06G015070.1	StnsLTP.044	PGSC0003DMG400025538	Soltu.DM.08G016090.1	StnsLTP.063

PGSC0003DMG400027400	Soltu.DM.11G025020.1	StnsLTP.121	PGSC0003DMG400025539	Soltu.DM.08G016100.1	StnsLTP.064
PGSC0003DMG402031237	Soltu.DM.10G018680.1	StnsLTP.105	PGSC0003DMG400043645	Soltu.DM.08G025530.1	StnsLTP.082
PGSC0003DMG400041307	Soltu.DM.10G006020.1	StnsLTP.097	PGSC0003DMG400043645	Soltu.DM.08G000480.1	StnsLTP.060
PGSC0003DMG400019325	Soltu.DM.06G034620.1	StnsLTP.053	PGSC0003DMG400032819	Soltu.DM.07G020060.1	StnsLTP.056
PGSC0003DMG400032250	Soltu.DM.06G015070.1	StnsLTP.044	PGSC0003DMG400022295	Soltu.DM.07G026820.1	StnsLTP.059
PGSC0003DMG400032260	Soltu.DM.06G015050.1	StnsLTP.043			

Note: This table lists the shared *nsLTP* genes between the previous report based on the DM v4.03 genome and our gene set annotated from the DM v6.1 genome. A total of 61 matched genes were identified. Genes newly annotated in this study are not listed here.
